# PGE2 activates EP4 in subchondral bone osteoclasts to regulate osteoarthritis

**DOI:** 10.1038/s41413-022-00201-4

**Published:** 2022-03-09

**Authors:** Wenhao Jiang, Yunyun Jin, Shiwei Zhang, Yi Ding, Konglin Huo, Junjie Yang, Lei Zhao, Baoning Nian, Tao P. Zhong, Weiqiang Lu, Hankun Zhang, Xu Cao, Karan Mehul Shah, Ning Wang, Mingyao Liu, Jian Luo

**Affiliations:** 1 Yangzhi Rehabilitation Hospital (Sunshine Rehabilitation Centre), Tongji University School of Medicine, Shanghai, PR China; 2 Shanghai Key Laboratory of Regulatory Biology, Institute of Biomedical Sciences and School of Life Sciences, East China Normal University, Shanghai, PR China; 3 Departments of Orthopaedic Surgery and Biomedical Engineering and Institute of Cell Engineering, The Johns Hopkins University School of Medicine, Baltimore, MD USA; 4 Department of Oncology and Metabolism, The University of Sheffield, Sheffield, UK

**Keywords:** Pathogenesis, Bone

## Abstract

Prostaglandin E2 (PGE2), a major cyclooxygenase-2 (COX-2) product, is highly secreted by the osteoblast lineage in the subchondral bone tissue of osteoarthritis (OA) patients. However, NSAIDs, including COX-2 inhibitors, have severe side effects during OA treatment. Therefore, the identification of novel drug targets of PGE2 signaling in OA progression is urgently needed. Osteoclasts play a critical role in subchondral bone homeostasis and OA-related pain. However, the mechanisms by which PGE2 regulates osteoclast function and subsequently subchondral bone homeostasis are largely unknown. Here, we show that PGE2 acts via EP4 receptors on osteoclasts during the progression of OA and OA-related pain. Our data show that while PGE2 mediates migration and osteoclastogenesis via its EP2 and EP4 receptors, tissue-specific knockout of only the EP4 receptor in osteoclasts (*EP4*^*LysM*^) reduced disease progression and osteophyte formation in a murine model of OA. Furthermore, OA-related pain was alleviated in the *EP4*^*LysM*^ mice, with reduced Netrin-1 secretion and CGRP-positive sensory innervation of the subchondral bone. The expression of platelet-derived growth factor-BB (PDGF-BB) was also lower in the *EP4*^*LysM*^ mice, which resulted in reduced type H blood vessel formation in subchondral bone. Importantly, we identified a novel potent EP4 antagonist, HL-43, which showed in vitro and in vivo effects consistent with those observed in the *EP4*^*LysM*^ mice. Finally, we showed that the Gαs/PI3K/AKT/MAPK signaling pathway is downstream of EP4 activation via PGE2 in osteoclasts. Together, our data demonstrate that PGE2/EP4 signaling in osteoclasts mediates angiogenesis and sensory neuron innervation in subchondral bone, promoting OA progression and pain, and that inhibition of EP4 with HL-43 has therapeutic potential in OA.

## Introduction

Osteoarthritis (OA) is a highly prevalent degenerative joint disease that affects over 300 million people worldwide^1,2^. In China, more than 8% of the population suffers from symptomatic OA, with an incidence rate of 62.2% in people over the age of 60 years and 80% in people aged 75 years and above^3^. The pathophysiology of OA involves the entire joint and is characterized by inflammation in the cartilage and synovium, degeneration of articular cartilage, formation of osteophytes, and sclerosis of subchondral bone^4–6^. Current therapeutic strategies primarily aim to mitigate the symptoms, and there are no satisfactory drugs or treatments that prevent or delay OA progression. In advanced stage OA, joint replacement surgery is the main strategy to improve patient quality of life and has a significant socioeconomic impact^7,8^. Therefore, a better understanding of the mechanisms underlying the pathogenesis of OA is urgently needed to inform novel treatment strategies.

The etiology of OA is multifactorial, and the risk of developing the disease increases with aging, obesity, joint trauma, increased mechanical loading, and genetic predisposition^9–13^. More recently, there has been increasing evidence to support subchondral bone homeostasis functions in the initiation and progression of OA^14–17^. Subchondral bone, which lies subjacent to the articular cartilage, absorbs, distributes, and transfers the mechanical loads experienced by the joints^6,18^. However, unstable mechanical loading, obesity and aging alter the subchondral bone microarchitecture to be more sclerotic, with increased remodeling and a higher frequency of bony cysts, which may precede cartilage damage^19–22^. Indeed, some studies have associated these changes in subchondral bone with an increase in the severity of cartilage damage and OA^23–25^.

In normal physiological conditions, the subchondral bone dynamically adjusts to the mechanical forces exerted on the joint via tightly coupled activity of osteoclasts and osteoblasts^26^. However, with abnormal loading, microfractures develop within the cartilage and underlying subchondral bone. At these subchondral bone microdamage sites, the bone remodeling process undergoes uncoupling, with increased osteoclast-mediated resorption and osteoblast-mediated uncoupled bone formation^27^. Osteoclastic bone resorption causes sharp increases in TGF-β1, which in turn recruits osteoprogenitor cells to the subchondral bone, leading to remineralization and sclerosis^28^. Furthermore, TGF-β1 promotes subchondral bone angiogenesis in early-stage OA^15^, including the formation of type H blood vessels, which closely associate with osteoprogenitor cells and are therefore osteogenic in nature^29^. These type H blood vessels further disrupt subchondral bone remodeling, alter the bone microarchitecture and lead to the formation of osteophytes and bone cysts^16,30^. Interestingly, osteoclast activity is also associated with increased sensory nerve innervation of the subchondral bone and hypersensitivity to pain in murine OA models^31,32^. Secretion of Netrin-1 by active osteoclasts mediates axonal growth in the subchondral bone^31,32^. The pivotal role of subchondral bone osteoclasts in OA progression makes them a suitable target for therapeutic invention. Indeed, there are trials to investigate the use of bisphosphonates, an inducer of osteoclast apoptosis, to target osteoclasts in OA^33–36^; however, the results thus far have been contradictory^37,38^. Thus, there is a need to explore other therapeutic options that target osteoclasts in OA.

Prostaglandin E2 (PGE2) is an important lipid mediator derived from the catalysis of arachidonic acid by cyclooxygenase enzymes (COX 1-2). PGE2 is the most abundant prostaglandin in the body, and under physiological conditions, it regulates various biological functions, including inflammation, blood pressure, fertility, and bone homeostasis^39–44^. In OA, COX-2 expression and PGE2 production have been associated with damaged articular cartilage, not normal cartilage, which suggests its role in disease pathogenesis^45^. Indeed, in a spontaneous murine model of OA, tissue-specific knockout of COX-2 in osteocytes, which abolished the production of PGE2 in subchondral bone, attenuated the progression of the disease^44^.

PGE2 exerts its complex biological effects via four E prostanoid (EP) rhodopsin-like G-protein coupled receptors (EP1–4)^46^. EP2 and EP4 are highly expressed in the skeletal system^47,48^, and EP4, in particular, plays an important regulatory role. *EP4* knockout mice lacked PGE2-induced bone formation, which was attributed to reduced osteoblast differentiation^49^. Furthermore, an EP4 agonist reduced bone loss in ovariectomized rats^49^. In OA, the role of the PGE2/EP4 signaling axis remains controversial, with studies reporting contradictory effects on proteoglycan synthesis and matrix degradation^50,51^. Moreover, the exact mechanisms by which PGE2 regulates subchondral bone homeostasis, specifically osteoclast function, in OA remain largely unknown.

In this study, we investigated how PGE2 acting via the EP4 receptor in osteoclasts, and not the EP2 receptor, may play an essential role in OA pathogenesis. Furthermore, we identified HL-43 as a novel potent EP4 antagonist that may have clinical applications for treating OA.

## Results

### The expression of EP4, but not EP2, is elevated in osteoclasts from osteoarthritic subchondral bone

**Fig. 1:**
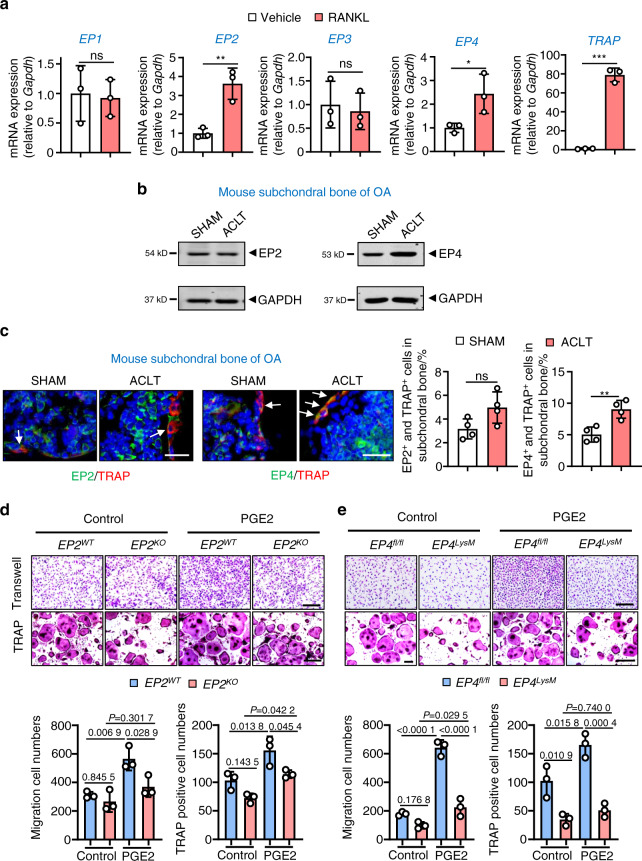
Expression of EP2 and EP4 in subchondral bone osteoclasts and the effects of PGE2 on osteoclasts with EP2 and EP4 deletion. **a** qRT-PCR analysis of the mRNA expression levels of *EP1*, *EP2*, *EP3*, *EP4*, and *Trap* in osteoclasts differentiated from BMMs exposed to 10 ng·mL^−1^ M-CSF and 50 ng·mL^−1^ RANKL for 5–6 days. Error bars are the mean ± s.d. *n* = 3. *P < 0.05, **P < 0.01 and ***P < 0.001, ns, not significant by unpaired two-tailed Student’s *t* test. **b** Representative images of the protein levels of EP2 and EP4 in mouse tibial subchondral bone tissue 2 weeks after sham or ACLT surgery. The experiments were performed with three biological replicates. **c** Representative images of IHF staining of EP2 or EP4 (green) and TRAP (red) in subchondral bone 2 weeks post-sham or ACLT surgery (left) and quantitative analysis (right). The white arrows indicate TRAP-positive osteoclasts expressing EP2 or EP4. Error bars are the mean ± s.d. *P < 0.05, ns, not significant by unpaired two-tailed Student’s *t* test. *n* = 4 for per group. Scale bars, 20 μm. **d** *EP2* deletion inhibits PGE2-induced migration and osteoclast differentiation. BMMs from the *EP2*^*WT*^ and littermate *EP2*^*KO*^ mice were used to generate osteoclasts by stimulation with 10 ng·mL^−1^ M-CSF and 50 ng·mL^−1^ RANKL and incubation with 100 nmol·L^−1^ PGE2. Representative image of cells from the Transwell migration assay (Transwell) and osteoclast differentiation assay (TRAP staining) (top) and the corresponding quantitative analysis (bottom). Error bars are the mean ± s.d. *n* = 3. Two-way ANOVA followed by Tukey’s *t* tests. Scale bars, 50 μm. **e** *EP2* deletion inhibits PGE2-induced migration and osteoclast differentiation. BMMs from the WT (*EP4*^*fl/fl*^) and littermate *EP4*^*fl/fl*^: *LysM-cre* (*EP4*^*LysM*^) mice were used to generate osteoclasts by stimulation with 10 ng·mL^−1^ M-CSF and 50 ng·mL^−1^ RANKL (5–6 days) and incubation with 100 nmol·L^−1^ PGE2. Representative image of cells from the Transwell migration assay (Transwell) and osteoclast differentiation assay (TRAP staining) (top) and the corresponding quantitative analysis (bottom). Error bars are the mean ± s.d. *n* = 3. Two-way ANOVA followed by Tukey’s *t* tests. Scale bars, 50 μm

To investigate the molecular mechanisms by which PGE2 may regulate subchondral bone osteoclasts, we determined the expression of the four PGE2 receptors (*EP1*–*EP4*) during osteoclastogenesis. Our results showed that the expression of *EP2* and *EP4*, but not *EP1* and *EP3*, is elevated in differentiated osteoclasts (Fig. 1a). We validated our findings in human osteoarthritic subchondral bone tissues (*n* = 17, Fig. S1a), which showed higher EP4 expression in 82.4% of samples (14/17) compared to the controls, with no changes in EP2 expression. These findings are also consistent with our ACLT-induced murine OA model (Fig. 1b), which is an extensively used animal model for research on osteoarthritic subchondral bone homeostasis. Furthermore, using immunofluorescence (IHF) double staining (EP2/EP4 with TRAP), we observed higher expression of EP4 but not EP2 in subchondral bone osteoclasts following ACLT surgery (Fig. 1c).

### PGE2 regulates migration and osteoclast differentiation through both EP2 and EP4

PGE2 increased migration and osteoclast differentiation from bone marrow-derived macrophages (BMMs) at nongrowth inhibitory concentrations (≤100 nmol·L^−1^ PGE2; Fig. S1b–d). To investigate the PGE2 receptors that may mediate these effects, we generated *EP2* knockout mice (*EP2*^*ko*^) (Fig. S2a) and deleted EP4 in myeloid cells by breeding *LysM-cre* mice with *EP4*^*fl/fl*^ mice (*EP4*^*LysM*^) (Fig. S3a). A significant reduction in EP2 or EP4 expression was observed in BMMs from the *EP2*^*ko*^ or *EP4*^*LysM*^ mice, indicating successful deletion of *EP2* or *EP4* in osteoclast lineage cells (Fig. S1e). We observed that knocking out *EP2* significantly reduced PGE2-induced osteoclast migration (*P* < 0.05) and differentiation (*P* < 0.05; Fig. 1d). A similar reduction in osteoclast migration (*P* < 0.001) and differentiation (*P* < 0.001) was also observed in the *EP4* knockout cells (Fig. 1e). Furthermore, commercially available specific antagonists of EP2 (PF-04418948) and EP4 (grapiprant) recapitulated the decrease in migration and osteoclast differentiation observed with the knockout cells (Fig. S1f). Interestingly, inhibition of EP4, either genetically or via an antagonist, had a more pronounced effect on migration and osteoclast differentiation than EP2 inhibition (Fig. S1f).

### EP4 deletion, but not EP2 deletion, inhibits OA progression in a murine model of OA

To investigate the role of EP2 and EP4 in OA progression, we used *EP2* knockout and *EP4* tissue-specific knockout mice for our ACLT-induced OA model. Our data suggest that *EP2* deficiency did not affect OA histological or subchondral bone parameters at 2 weeks (Fig. S2b) or 8 weeks (Fig. S2c) after ACLT surgery. The formation of osteophytes and activation of osteoclasts were also not different between the *EP2* knockout and WT mice after ACLT surgery (Fig. S2b, c).

**Fig. 2:**
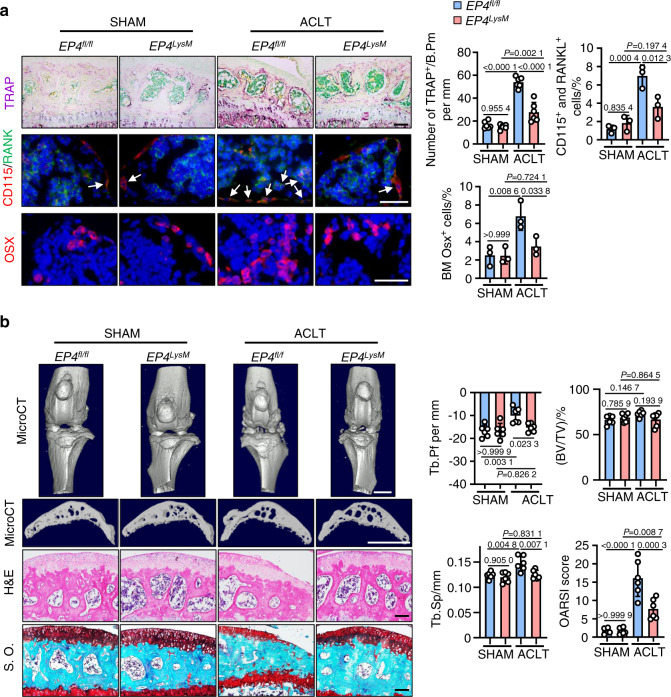
EP4 deletion in osteoclasts inhibits OA progression in a murine OA model. **a** Representative images of TRAP staining and IHF staining of Osterix (Osx), CD115 (red), and RANK (green) in the subchondral bone of the *EP4*^*fl/fl*^ or littermate *EP4*^*LysM*^ mice post-ACLT surgery (top) and quantitative analysis (bottom). TRAP staining, IHF staining of CD115 and RANK, and IHC staining of Osx were performed 2 weeks after ACLT surgery. The white arrow indicates CD115 and RANK double-positive osteoclast precursors. Error bars are the mean ± s.d. Two-way ANOVA followed by Tukey’s *t* tests. *n* = 6 for TRAP staining, *n* = 3 for CD115 and RANK IHF staining. Scale bars, 50 μm (TRAP staining), 20 μm (CD115 and RANK IHF staining), and 20 μm (Osx IHF staining). **b** Representative 3D reconstructed microCT images, H&E staining, and safranin O-fast green (S.O.) staining of sagittal sections of articular cartilage of the *EP4*^*fl/fl*^ or littermate *EP4*^*LysM*^ mice 8 weeks after ACLT surgery (left). Quantitative analysis of structural parameters of subchondral bone, including Tb.Pf, Tb.Sp and BV/TV. OARSI scores based on the S.O. staining histology analysis (right). Error bars are the mean ± s.d. *n* = 6 for each group. Two-way ANOVA followed by Tukey’s *t* tests. Scale bars, 1 mm (microCT), 50 μm (S.O.) and 50 μm (H&E)

In contrast, tissue-specific knockout of *EP4* in myeloid cells *(EP4*^*LysM*^*)* mildly altered the subchondral bone microarchitecture and protected against OA-induced changes in trabecular bone 2 weeks post-ACLT surgery (*P* < 0.05; Fig. S3b) compared to those of the *EP4*^*fl/fl*^ controls. Furthermore, a remarkable reduction in osteoclast precursor (CD115^+^/RANK^+^ monocyte) recruitment (*P* < 0.05) and osteoclast activity (TRAP activity, *P* < 0.001) was observed in the subchondral bone of the *EP4*^*LysM*^ mice compared to the *EP4*^*fl/fl*^ control mice at this time point (Fig. 2a). Osterix-positive osteoprogenitor cells, which were increased in the subchondral bone marrow of the *EP4*^*fl/fl*^ mice (*P* < 0.01) post-ACLT, remained at basal levels in the *EP4*^*LysM*^ mice (Fig. 2a). At a later time point of 8 weeks post-ACLT surgery, histological and subchondral bone parameters were markedly better in the *EP4*^*LysM*^ mice than in the *EP4*^*fl/fl*^ control mice and were reflected in their improved OARSI scores (Fig. 2b). Moreover, at 8 weeks post-ACLT surgery, MMP13- and type X collagen (COLX)-positive articular chondrocytes, which are markers for cartilage degeneration, were lower in the *EP4*^*LysM*^ mice than in the littermate *EP4*^*fl/fl*^ control mice (Fig. S3c). Taken together, the data suggest that PGE2 acting via EP4, and not EP2, may mediate OA progression in an ACLT surgery-induced murine OA model.

### *EP4* knockout in osteoclasts reduces pain in OA by suppressing Netrin-1 secretion and recruiting CGRP-positive sensory neurons to subchondral bone

**Fig. 3:**
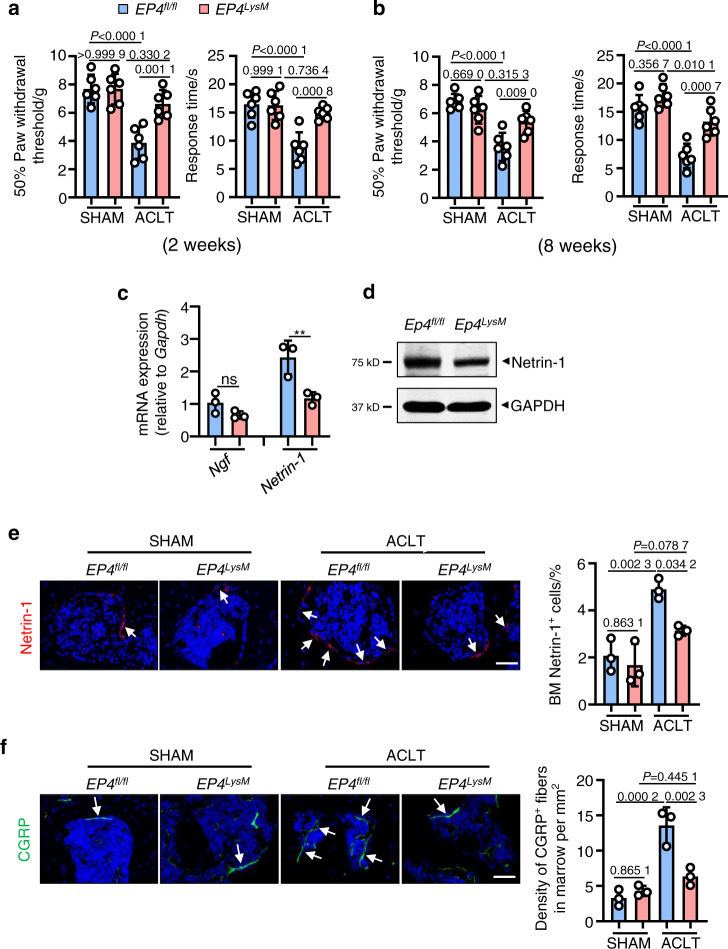
EP4 deletion in osteoclasts reduces pain in OA by suppressing Netrin-1 secretion and CGRP-positive sensory neuron recruitment to subchondral bone. Von Frey assay and thermal hyperalgesia test conducted at the right hind paw of the *EP4*^*fl/fl*^ or littermate *EP4*^*LysM*^ mice 2 weeks (**a**) or 8 weeks (**b**) after ACLT surgery. Error bars are the mean ± s.d. Two-way ANOVA followed by Tukey’s t tests. *n* = 6 for each group. **c** qRT-PCR analysis of the mRNA levels of *Ngf* and *Netrin-1* in osteoclasts generated from BMMs stimulated with 10 ng·mL^−1^ M-CSF and 50 ng·mL^−1^ RANKL and incubated with 100 nmol·L^−1^ PGE2 for 5 days. The experiments were performed with three biological replicates. Error bars are the mean ± s.d. **P < 0.01 and ns, not significant by one-way ANOVA followed by Tukey’s *t* tests. **d** Representative images of Netrin-1 protein by western blotting of osteoclasts generated using BMMs from the *EP4*^*fl/fl*^ or littermate *EP4*^*LysM*^ mice stimulated with 10 ng·mL^−1^ M-CSF and 50 ng·mL^−1^ RANKL and incubated with 100 nmol·L^−1^ PGE2 for 5 days. The experiments were performed with three biological replicates. Representative images of IHF staining of Netrin-1 (**e**, red) and CGRP-positive sensory nerve fibers (**f**, red) in the subchondral bone of the *EP4*^*fl/fl*^ or littermate *EP4*^*LysM*^ mice 2 weeks after ACLT surgery (left) and corresponding quantitative analysis (right). The white arrows indicate Netrin-1 or CGRP IHF signals. Error bars are the mean ± s.d. Two-way ANOVA followed by Tukey’s t tests. *n* = 3 for each group. Scale bars, 20 μm

One of the common symptoms of OA is pain, and here, we assessed mechanical (von Frey filaments) and thermal nociception hyperalgesia in mice compared to that of the *EP4*^*fl/fl*^ controls. We observed that at both the 2- and 8-week time points, the paw withdrawal threshold of the *EP4*^*LysM*^ mice remained at basal sham-operated levels compared to that of the *EP4*^*fl/fl*^ mice, which exhibited a significantly lower threshold following ACLT surgery (*P* < 0.001; Fig. 3a and b).

Subchondral bone osteoclasts have been implicated in recruiting sensory nerves via secretion of nerve growth factor (NGF) and Netrin-1 and thus mediate pain in OA^31,52^. We found that PGE2-induced expression of *Netrin-1* was significantly lower at the transcript level (*P* < 0.01; Fig. 3c) in osteoclasts from the *EP4*^*LysM*^ mice. Furthermore, western blotting confirmed lower expression of Netrin-1 in osteoclasts from the *EP4*^*LysM*^ mice than in those generated from the *EP4*^*fl/fl*^ mice (Fig. 3d). The results were corroborated in an in vivo setting, with Netrin-1 expression being significantly lower in the subchondral bone marrow of the *EP4*^*LysM*^ mice following ACLT (*P* < 0.05) than in that of the *EP4*^*fl/fl*^ mice (Fig. 3e). The CGRP-positive sensory neurons that may mediate OA-related pain were also significantly lower in the subchondral bone marrow of the *EP4*^*LysM*^ mice than in that of the *EP4*^*fl/fl*^ mice (*P* < 0.01; Fig. 3f). Thus, the data suggest that the reduction in OA-related pain observed in the *EP4*^*LysM*^ mice may partly be due to the reduced expression of Netrin-1 and a subsequent reduction in CGRP-positive sensory neuron recruitment to subchondral bone.

### *EP4* knockout in osteoclasts reduces type H blood vessels in subchondral bone via inhibition of PDGF-BB secretion

**Fig. 4:**
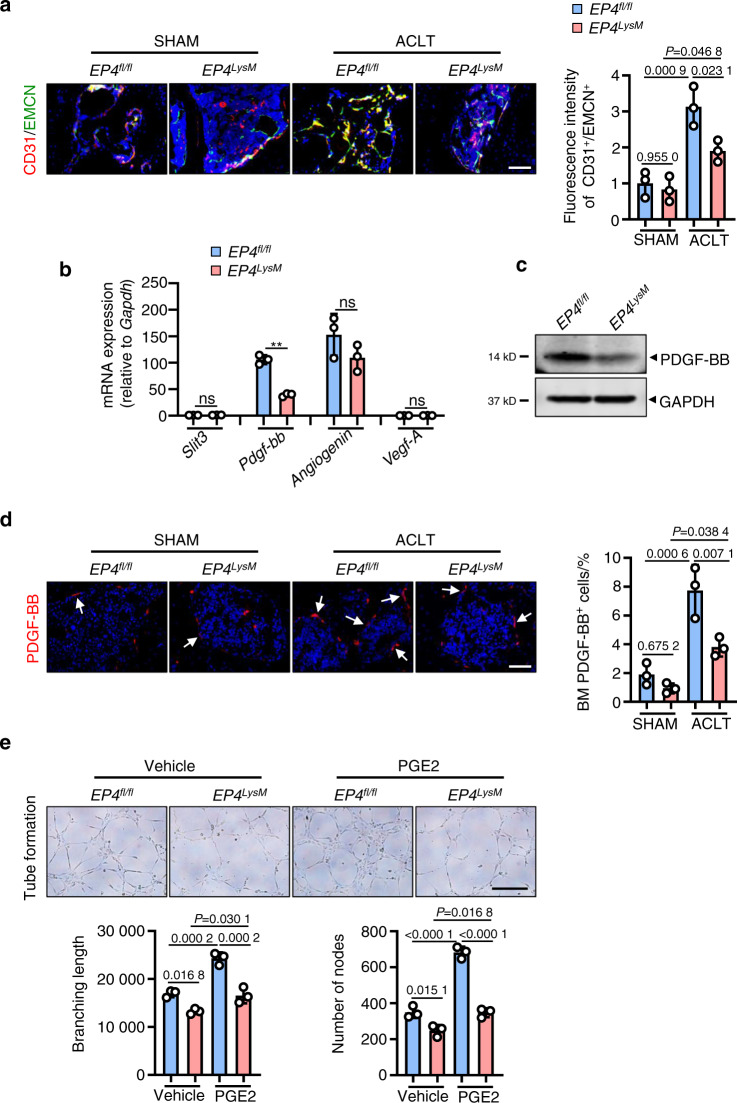
EP4 deletion in osteoclasts reduces type H blood vessels in subchondral bone via inhibition of PDGF-BB secretion. **a** Representative images for IHF staining of EMCN (green) and CD31 (red) in subchondral bone of the *EP4*^*fl/fl*^ or littermate *EP4*^*LysM*^ mice 2 weeks after ACLT surgery (left) and corresponding quantitative analysis (right). Error bars are the mean ± s.d. Two-way ANOVA followed by Tukey’s *t* tests. *n* = 3 for each group. Scale bars, 20 μm. **b** qRT-PCR analysis of the mRNA levels of *Slit3*, *Pdgf-bb*, *Angiogenin*, and *Vegf-A* in osteoclasts generated from BMMs of the *EP4*^*fl/fl*^ or littermate *EP4*^*LysM*^ mice stimulated with 10 ng·mL^−1^ M-CSF and 50 ng·mL^−1^ RANKL and incubated with 100 nmol·L^−1^ PGE2 for 24 h. Error bars are the mean ± s.d. *n* = 3. **P < 0.01 and ns, not significant by one-way ANOVA followed by Tukey’s *t* tests. **c** Representative images of PDGF-BB protein expression by western blotting of osteoclasts generated using BMMs from the *EP4*^*fl/fl*^ or littermate *EP4*^*LysM*^ mice stimulated with 10 ng·mL^−1^ M-CSF and 50 ng·mL^−1^ RANKL and incubated with 100 nmol·L^−1^ PGE2 for 3 days. The experiments were performed with three biological replicates. **d** Representative images of IHF staining of PDGF-BB (red) in the subchondral bone of the *EP4*^*fl/fl*^ or littermate *EP4*^*LysM*^ mice 2 weeks after ACLT surgery (left) and corresponding quantitative analysis (right). The white arrows indicate PDGF-BB IHF signals. Error bars are the mean ± s.d. Two-way ANOVA followed by Tukey’s *t* tests. *n* = 3 for each group. Scale bars, 20 μm. **e** Representative images of the HUVEC tube formation assay (left). BMMs isolated from the *EP4*^*fl/fl*^ or littermate *EP4*^*LysM*^ mice were stimulated with 10 ng·mL^−1^ M-CFS, 60 ng·mL^−1^ RANKL and 100 nmol·L^−1^ PGE2 for 3 days. Subsequently, conditioned medium from osteoclasts was used to treat HUVECs for 4 h. The branching length and node numbers of the HUVEC tubes were quantitated (right). Error bars are the mean ± s.d. *n* = 3. Two-way ANOVA followed by Tukey’s t tests. Scale bars, 50 μm

Increases in subchondral bone angiogenesis and blood vessel branching are clinical features of OA^53^. Our data suggest that ACLT surgery in the *EP4*^*fl/fl*^ mice increases type H blood vessels (CD31^high^/Emcn^high^), which are known to disrupt subchondral bone homeostasis^16,30^ (*P* < 0.001; Fig. 4a). The induction of type H blood vessels was significantly blunted in the *EP4*^*LysM*^ mice (Fig. 4a).

Osteoclast-secreted cytokines, including VEGF-A^54^, PDGF-BB^16^, SLIT3^55^ and angiogenin^56^, can regulate type H blood vessel production and may have a significant role in OA progression. Here, we observed that of these cytokines, only PDGF-BB was differentially expressed between the *EP4*^*LysM*^ and *EP4*^*fl/fl*^ mice following PGE2 treatment at both the transcript and protein levels (Fig. 4b, c). It has been reported that PDGF-BB secreted by osteoclast precursors can promote subchondral bone angiogenesis and osteogenesis and facilitate osteoarthritis progression^29^. Furthermore, IHF staining of subchondral bone marrow showed reduced PDGF-BB-positive cells in the *EP4*^*LysM*^ mice compared to the *EP4*^*fl/fl*^ mice 2 weeks post-ACLT surgery (*P* < 0.01; Fig. 4d). To investigate whether this reduction in angiogenesis is mediated via secreted factors by osteoclasts from *EP4*^*LysM*^, we performed tube formation assays using HUVECs treated with conditioned media (CM) from the *EP4*^*LysM*^-derived osteoclast precursor cells. Our data show that CM from the *EP4*^*LysM*^-derived osteoclast precursors reduces the tube branch length and nodes compared to those of the *EP4*^*fl/fl*^-derived cells (*P* < 0.05; Fig. 4e). This effect was also more pronounced for CM collected from osteoclast precursors treated with PGE2 (*P* < 0.001; Fig. 4e).

### Identification of a novel potent EP4 antagonist for PGE2-induced migration and osteoclast differentiation

**Fig. 5:**
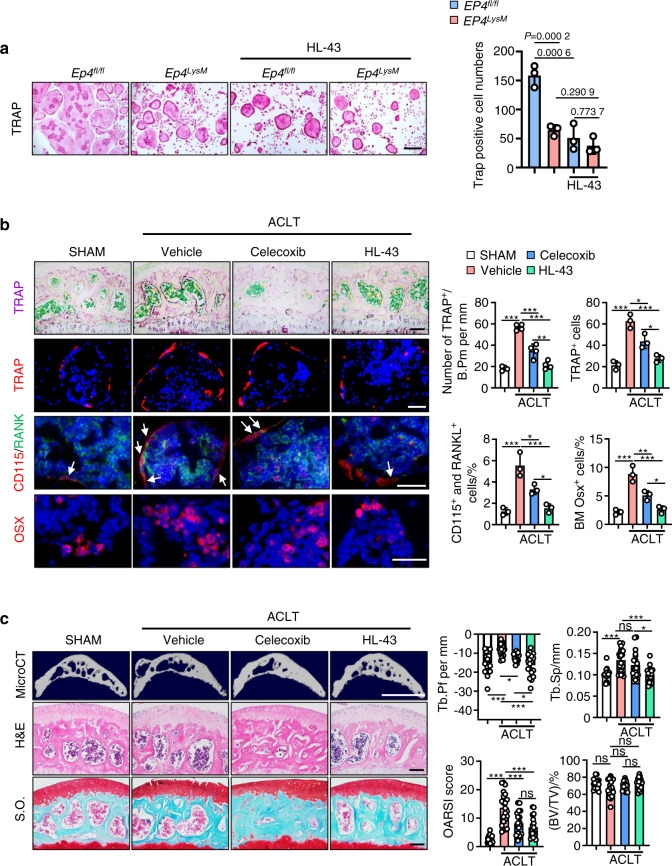
The EP4 antagonist HL-43 inhibits OA progression in a murine OA model. **a** HL-43 inhibits PGE2-induced osteoclast differentiation. BMMs from the *EP4*^*fl/fl*^ mice and littermate *EP4*^*fl/fl*^: *LysM-cre* mice (*EP4*^*LysM*^) were used to generate osteoclasts by stimulation with 10 ng·mL^−1^ M-CSF and 50 ng·mL^−1^ RANKL and incubation with 100 nmol·L^−1^ PGE2 in the presence and absence of HL-43 (10 μmol·L^−1^). Representative images of TRAP staining are on the left, and the corresponding quantification is on the right. Error bars are the mean ± s.d. *n* = 3. Two-way ANOVA followed by Tukey’s t tests. Scale bars, 50 μm. **b** Representative images of TRAP staining and IHF staining of TRAP (red), Osx (red), CD115 (red), and RANK (green) in the subchondral bone of the WT mice orally treated with the FDA-approved OA pain drug celecoxib (30 mg·kg^−1^) or HL-43 (30 mg·kg^−1^) 2 weeks after ACLT surgery (left) and quantitative analysis (right). The white arrows indicate CD115 and RANK double-positive osteoclast precursors. Error bars are the mean ± s.d. *P < 0.05, **P < 0. 01 and ***P < 0.001 by one-way ANOVA followed by Tukey’s t tests. *n* = 4 for TRAP staining, *n* = 3 for TRAP IHF staining, *n* = 3 for CD115 and RANK IHF staining, *n* = 3 for Osx IHC staining. Scale bars, 50 μm (TRAP staining), 20 μm (TRAP IHF staining), 20 μm (CD115 and RANK IHF staining) and 20 μm (Osx IHF staining). **c** Representative 3D reconstructed microCT images, H&E staining, and S.O. staining of articular cartilage of the WT mice treated with celecoxib or HL-43 8 weeks after ACLT surgery (left). Mice were orally treated daily with celecoxib (30 mg·kg^−1^) or HL-43 (30 mg·kg^−1^) for 8 weeks. Quantitative analysis of structural parameters of subchondral bone, including Tb.Pf, Tb.Sp, and BV/TV. OARSI scores based on S.O. staining histology analysis (right). Error bars are the mean ± s.d. *P < 0.05, **P < 0.01 and ***P < 0.001, ns, not significant by one-way ANOVA followed by Tukey’s *t* tests. *n* = 17 for the sham group, *n* = 21 for the vehicle-treated group, *n* = 18 for the celecoxib-treated group, *n* = 18 for the HL-43-treated group. Scale bars, 1 mm (microCT), 50 μm (H&E) and 50 μm (S.O.)

To identify a potent small molecule EP4 antagonist with therapeutic potential in OA, we screened a class of small molecule compounds with 1H-1,2,3-triazole-based structures^57^, with osteoclast differentiation as the primary outcome. HL-43, which refers to compound 43 in the library, was the most potent inhibitor of PGE2-induced osteoclast differentiation (Fig. S4a). We confirmed that HL-43 mediated its effects via EP4 by assessing osteoclast differentiation from the *EP4*^*fl/fl-*^ and *EP4*^*LysM*^-derived cells (Fig. 5a). Our results show that HL-43 treatment significantly reduced the number of osteoclasts generated from the *EP4*^*fl/fl-*^derived cells, with no effect on the *EP4*^*LysM*^-derived cells (Fig. 5a). A dose-response curve with HL-43 for osteoclast differentiation gave an IC_50_ of 1.215 μmol·L^−1^ and an IC_50_ of 0.239 μmol·L^−1^ for migration (Fig. S4b, c).

### HL-43, an EP4 antagonist, inhibits OA progression in a murine model of OA

To investigate the therapeutic potential of HL-43 in OA, we tested its effects on OA progression and subchondral bone parameters in our ACLT-induced murine OA model. Celecoxib, a commonly used FDA-approved COX-2 inhibitor for the clinical treatment of OA, was used in a comparison of the efficacy of HL-43. Following two weeks of oral administration of HL-43, mice had a dramatically lower number of TRAP-positive subchondral bone osteoclasts post-ACLT surgery than those that received the vehicle controls (*P* < 0.001; Fig. 5b) and celecoxib treatment (*P* < 0.05; Fig. 5b). HL-43 significantly decreased the numbers of CD115 and RANK double-positive osteoclast precursors and Osterix-positive osteoprogenitor cells in the subchondral bone compared to vehicle (*P* < 0.001) and celecoxib (*P* < 0.05; Fig. 5b).

While no significant differences in bone microarchitecture and histological parameters were observed two weeks post-ACLT surgery (Fig. S5a), the changes in the subchondral bone populations following HL-43 treatment were reflected at 8 weeks, with improved bone microarchitecture and histological parameters and reduced osteophytes and articular cartilage defects observed post-ACLT surgery (Fig. 5c). The expression of MMP13 and COLX was also lower after ACLT surgery than after vehicle treatment (*P* < 0.001; Fig. S5b), with no differences observed compared to those after celecoxib treatment.

Furthermore, celecoxib is associated with several severe side effects to the cardiovascular system, kidneys, intestines, stomach and liver^58–61^. Indeed, red blood cell infiltration was observed in the gastric tissues of the mice treated with celecoxib, which was absent in the HL-43-treated group (Fig. S5c). Celecoxib was also more cytotoxic to BMMs (IC_50_: 50.64 μmol·L^−1^) and BMSCs (IC_50_: 41 μmol·L^−1^) than HL-43 (IC_50_ > 300 μmol·L^−1^ for both; Fig. S5d), which may have implications for adverse reactions. Taken together, our data suggest that the EP4 antagonist HL-43 could reduce OA progression by targeting subchondral bone osteoclast activity, retaining the bone microarchitecture and reducing osteophyte formation, with minimal side effects.

### HL-43 reduces pain in OA by suppressing Netrin-1 secretion and recruiting CGRP-positive sensory neurons to subchondral bone

**Fig. 6:**
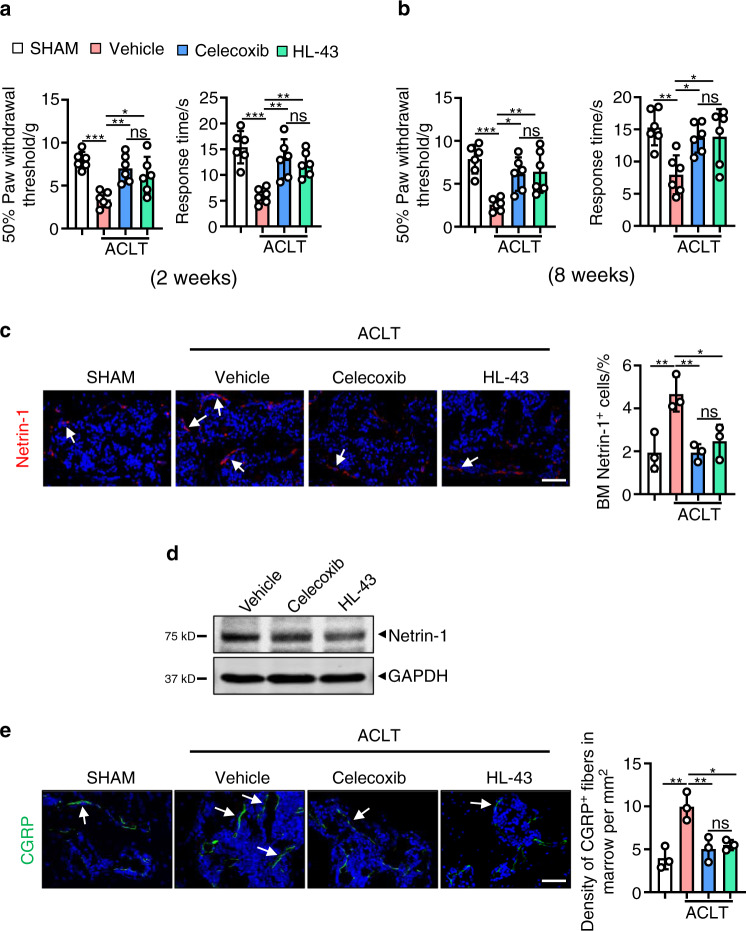
HL-43 reduces pain in OA by suppressing Netrin-1 secretion by osteoclasts and CGRP-positive sensory neuron recruitment to subchondral bone. Von Frey assay and thermal hyperalgesia test conducted at the right hind paw of the WT mice treated with celecoxib (30 mg·kg^−1^) or HL-43 (30 mg·kg^−1^) for 2 weeks (**a**) or 8 weeks (**b**) after ACLT surgery. Error bars are the mean ± s.d. **P < 0.01 and ***P < 0.001, ns, not significant by one-way ANOVA followed by Tukey’s *t* tests. *n* = 6 for each group. **c** Representative images of IHF staining of Netrin-1 (red) in the subchondral bone of the WT mice orally treated with celecoxib (30 mg·kg^−1^) or HL-43 (30 mg·kg^−1^) 2 weeks post-ACLT surgery (left) and quantitative analysis (right). The white arrows indicate Netrin-1 IHF signals. Error bars are the mean ± s.d. *P < 0.05, **P < 0.01 and ***P < 0.001, ns, not significant by one-way ANOVA followed by Tukey’s *t* tests. *n* = 3 for each group. Scale bars, 20 μm. **d** Representative images of Netrin-1 protein expression by western blotting for osteoclasts generated using BMMs from WT mice stimulated with 10 ng·mL^−1^ M-CSF and 50 ng·mL^−1^ RANKL. The cells were incubated with 100 nmol·L^−1^ PGE2 with or without celecoxib (10 μmol·L^−1^) or HL-43 (10 μmol·L^−1^) for 5 days. The experiments were performed with three biological replicates. **e** Representative images of IHF staining of CGRP-positive sensory nerve fibers (red) in the subchondral bone of the WT mice orally treated with celecoxib (30 mg·kg^−1^) or HL-43 (30 mg·kg^−1^) 2 weeks post-ACLT surgery (left) and quantitative analysis (right). The white arrows indicate CGRP IHF signals. Error bars are the mean ± s.d. **P < 0.01 and ***P < 0.001, ns, not significant by one-way ANOVA followed by Tukey’s *t* tests. *n* = 3 for each group. Scale bars, 20 μm

Similar to data obtained with the *EP4*^*LysM*^ mice, we observed that at both the 2- and 8-week time points, the paw withdrawal threshold following HL-43 treatment was higher with mechanical nociception and thermal nociception hyperalgesia than that of the vehicle group following ACLT surgery (*P* < 0.05; Fig. 6a and b). Netrin-1 expression was also significantly lower in subchondral bone marrow in the HL-43-treated group than in the vehicle-treated group (*P* < 0.05; Fig. 6c) and in primary osteoclasts in vitro (Fig. 6d). CGRP-positive sensory neurons in the subchondral bone marrow were also lower in the HL-43-treated mice than in the vehicle-treated mice (*P* < 0.05; Fig. 6e). No significant differences were observed in mechanical and thermal nociceptive hyperalgesia or Netrin-1- or CGRP-positive sensory neuron expression between the HL-43 and celecoxib treatment groups at either time point (Fig. 6a–e).

### HL-43 reduces type H blood vessels in subchondral bone via inhibition of PDGF-BB secretion by osteoclasts

**Fig. 7:**
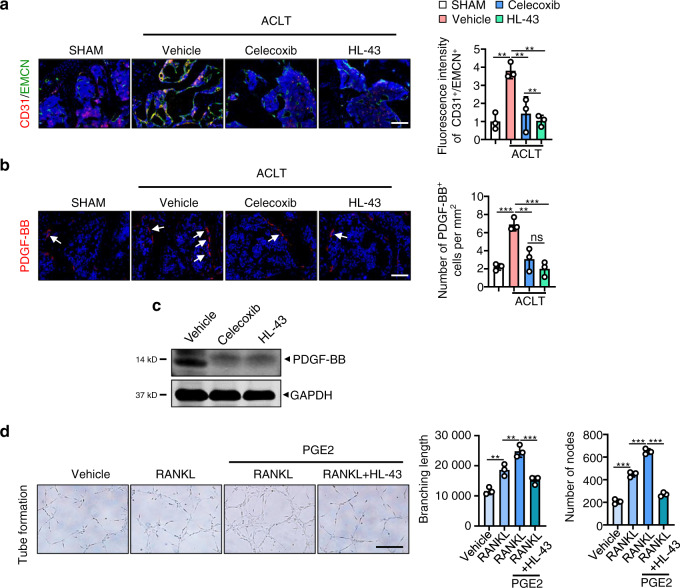
HL-43 reduces type H blood vessels in subchondral bone via inhibition of PDGF-BB secretion by osteoclasts. Representative images for IHF staining of **a** EMCN (green) and CD31 (red) type H vessels and **b** PDGF-BB (red) in subchondral bone of the WT mice orally treated with celecoxib (30 mg·kg^−1^) or HL-43 (30 mg·kg^−1^) 2 weeks post-ACLT surgery (left) and quantitative analysis (right). The white arrows indicate PDGF-BB IHF signals. Error bars are the mean ± s.d. *P < 0.05, **P < 0.01 and ***P < 0.001 by one-way ANOVA followed by Tukey’s *t* tests. *n* = 3 for each group. Scale bars, 20 μm. **c** Representative images of PDGF-BB protein expression by western blotting for osteoclasts generated using BMMs from WT mice stimulated with 10 ng·mL^−1^ M-CSF and 50 ng·mL^−1^ RANKL. The cells were incubated with 100 nmol·L^−1^ PGE2 with or without celecoxib (10 μmol·L^−1^) or HL-43 (10 μmol·L^−1^) for 3 days. The experiments were performed with three biological replicates. **d** Representative images of the HUVEC tube formation assay (left). BMMs isolated from the WT mice were stimulated with either 10 ng·mL^−1^ M-CSF alone or with 60 ng·mL^−1^ RANKL. Other treatment conditions included BMMs stimulated with M-CSF and RANKL, 100 nmol·L^−1^ PGE2, with and without HL-43 (10 μmol·L^−1^) for 3 days. Subsequently, conditioned medium from osteoclasts was used to treat HUVECs for 4 h. The branching length and node numbers of the HUVEC tubes were quantitated (right). Error bars are the mean ± s.d. *P < 0.05, **P < 0.01 and ***P < 0.001 by one-way ANOVA followed by Tukey’s *t* tests. *n* = 3 for each group. Scale bars, 50 μm

HL-43 treatment in the murine OA model reduced the presence of type H blood vessels (CD31^high^/Emcn^high^) in the subchondral bone compared to that of the vehicle-treated group (*P* < 0.05; Fig. 7a). Analysis of subchondral bone via IHF staining and primary osteoclasts via western blotting showed reduced expression of PDFG-BB following HL-43 treatment (*P* < 0.001; Fig. 7b and c). Tube branching length and node numbers were also lower in the HUVECs exposed to conditioned medium from the PGE2-induced osteoclasts treated with HL-43 (*P* < 0.001; Fig. 7d).

### The PGE2/EP4 signaling axis regulates migration and osteoclast differentiation via Gαs/PI3K/AKT/MAPK activation

**Fig. 8:**
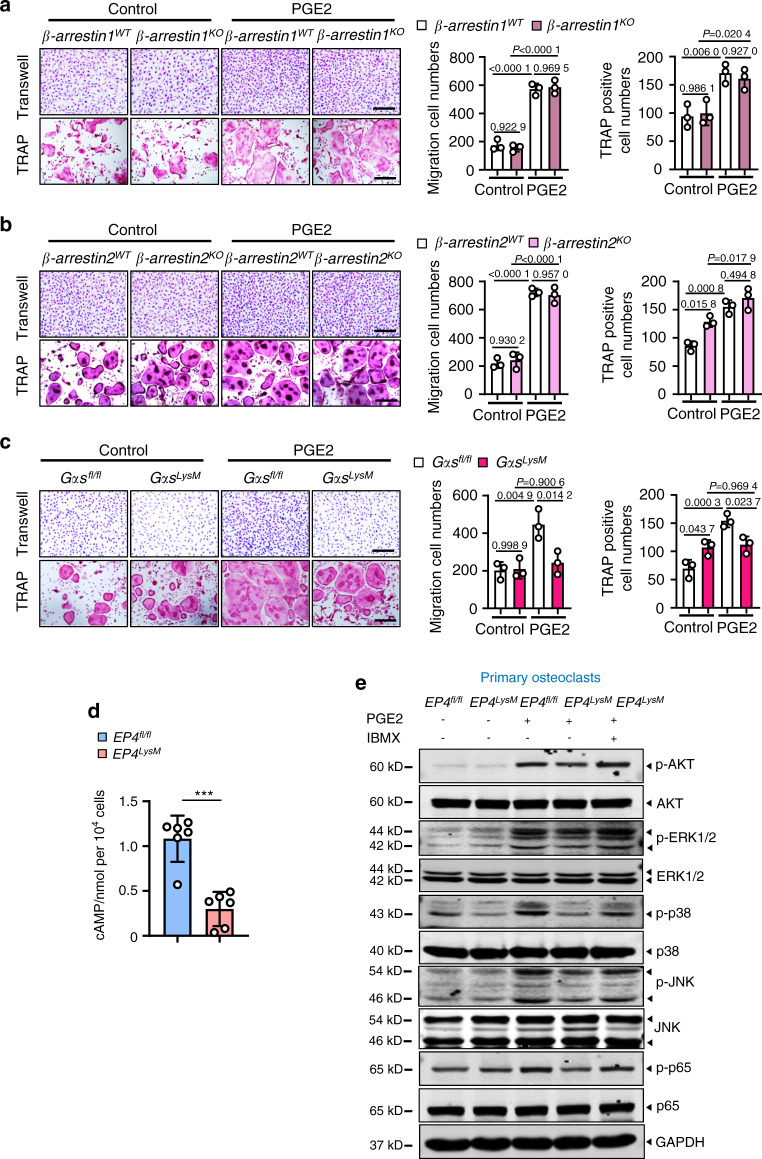
PGE2 regulates migration and osteoclast differentiation through the Gαs/PI3K/AKT/MAPK signaling pathways downstream of EP4. PGE2 regulates migration and osteoclast differentiation independent of β-arrestin1 (**a**) and β-arrestin2 (**b**) but via Gαs (**c**). BMMs from the WT and littermate *β-arrestin1-* and *β-arrestin2-*knockout mice were used to generate osteoclasts by stimulation with 10 ng·mL^−1^ M-CSF and 50 ng·mL^−1^ RANKL and incubation with 100 nmol·L^−1^ PGE2. For Gαs experiments, osteoclasts were generated from the GMMs of the *Gαs*^*fl/fl*^ controls and *Gαs*^*fl/fl*^; *LysM-cre* (*Gαs*^*LysM*^) mice. A differentiation assay (TRAP staining) (left) and the corresponding quantitative analysis (right). Scale bars, 50 μm. Error bars are the mean ± s.d. *n* = 3. Two-way ANOVA followed by Tukey’s *t* tests. Scale bars, 50 μm. **d** cAMP production was measured via ELISAs for osteoclasts derived from the *Ep4*^*fl/fl*^ and *Ep4*^*LysM*^ mice. BMMs from the two mouse strains were isolated and stimulated with 10 ng·mL^−1^ M-CSF and 50 ng·mL^−1^ RANKL to differentiate into osteoclasts and incubated with 100 nmol·L^−1^ PGE2 for 30 min prior to cAMP measurements. Error bars are the mean ± s.d. ***P < 0.001 by unpaired two-tailed Student’s *t* test. The experiment was performed with three biological replicates. **e** Representative images of the indicated protein expression by western blotting for osteoclasts generated using BMMs of *Ep4*^*fl/fl*^ and *Ep4*^*LysM*^ mice. The cells were treated either with osteoclastogenic media (10 ng·mL^−1^ M-CSF and 50 ng·mL^−1^ RANKL) alone, with PGE2 (100 nmol·L^−1^), or PGE2 with IBMX (1 mmol·L^−1^) for 3 h. The experiments were performed with three biological replicates

Previous reports in several cells and tissues have suggested that PGE2/EP4 signaling may activate the Gαs or β-arrestin pathways^62–64^. BMMs from *β-arrestin1* and *β-arrestin2* knockout mice did not show any changes in the ability of PGE2 to induce osteoclast differentiation and migration compared to the WT cells (Fig. 8a, b). However, deletion of *Gαs* from these precursors impaired their migration (*P* < 0.05) and differentiation to osteoclasts following PGE2 treatment (*P* < 0.05; Fig. 8c). PGE2/EP4 signaling via Gαs induced cAMP production and protein kinase A signaling downstream activation^64^. Here, we observed that in osteoclasts from the *EP4*^*LysM*^ mice, PGE2 treatment resulted in markedly lower cAMP production than osteoclasts from the *EP4*^*fl/fl*^ mice (*P* < 0.001; Fig. 8d). Moreover, using a phosphoproteome antibody array (157 phosphorylation antibodies) to identify differential pathway activation with PGE2 in these cells, we found alterations in cAMP, PI3K/AKT, and MAPK signaling (Fig. S6).

As the activation of PI3K/AKT signaling pathways was remarkably different between the *EP4*^*LysM*^ and *EP4*^*fl/fl*^ cells and its role in osteoclast differentiation is well established^65^, this pathway was our primary choice for further investigations. Indeed, PGE2-induced phosphorylation of AKT was dampened in cells from the *EP4*^*LysM*^ mice but was rescued in the presence of 3-isobutyl-1-methylxanthine (IBMX), which protects against cAMP degradation (Fig. 8e). Furthermore, PGE2-induced migration (*P* < 0.05; Fig. S7a) and differentiation (*P* < 0.05; Fig. S7a) of the WT mice were significantly suppressed following treatment with an AKT inhibitor (GSK2141795).

The phosphoproteome antibody array also identified MAPK signaling, a pathway downstream of PI3K/AKT^66^, to be altered between the *EP4*^*LysM*^ and *EP4*^*fl/fl*^ cells. We observed suppression of the p-ERK, p-38 and p-JNK MAPKs in cells lacking EP4 following PGE2 treatment (Fig. 8e), but this change was reversed in the presence of IBMX. Additionally, AKT inhibition via GSK2141795 in the presence of PGE2 suppressed the phosphorylation of these MAPKs compared to vehicle treatment (Fig. S7b). Inhibition of MAPKs also resulted in a reduction in PGE2-induced migration and osteoclast differentiation (Fig. S7c).

Finally, we investigated NF-κB signaling, which is downstream of PI3K/AKT activation and mediates osteoclastogenesis^67^. Interestingly, NF-κB signaling also regulates PDGF-BB expression and may have implications for osteoclast-mediated type H vessel formation in OA. Neferine, an inhibitor of NF-κB signaling, strikingly suppressed PGE2-induced PDGF-BB expression in osteoclasts from the WT mice (Fig. S7d). Moreover, NF-κB (p65) phosphorylation was suppressed in cells lacking EP4 following PGE2 treatment (Fig. 8e), which was rescued with IBMX.

Thus, the data here suggest that inhibiting PGE2/EP4 signaling occurs via the Gαs/cAMP/PI3K/AKT/MAPK axis and that PGE2/EP4/cAMP signaling decreases the expression of PDGF-BB through the NF-κB pathway.

## Discussion

Alterations in the subchondral bone microarchitecture, usually caused by aging or mechanical trauma, are a primary risk factor for OA^68^. In comparison to normal cartilage, osteoarthritic tissues express elevated levels of COX-2 and consequently have higher levels of PGE2^45^. PGE2 plays an important role in OA, but how it regulates subchondral bone homeostasis, specifically osteoclast function, remains unclear. Here, we observed elevated expression of the PGE2 receptor EP4 in osteoclasts from subchondral bone. Genetic deletion of the EP4 receptor in osteoclasts or using a specific inhibitor reduced OA progression in a murine model. Furthermore, this effect was accompanied by lower recruitment of type H blood vessels and CGRP-positive sensory neurons in the subchondral bone, with implications for disease progression and pain. We also identified the primary signaling pathway triggered in subchondral bone osteoclasts following activation of EP4 via PGE2. Finally, we identified HL-43 as a candidate small molecule for the treatment of OA; these molecules had better efficacy in improving subchondral bone microarchitecture than celecoxib (clinically approved COX-2 inhibitor) and fewer side effects.

While PGE2 is a known regulator of bone homeostasis, the direct effects of PGE2 on osteoclast activity remain controversial, with studies showing both stimulatory and inhibitory effects^69,70^. The migration of osteoclast precursors to the site of bone remodeling and differentiation into multinucleated osteoclasts are the keys to osteoclast function. Here, using genetic deletion, we show that PGE2 acting via the EP2 and EP4 receptors can regulate migration and osteoclast differentiation, consistent with a previous finding by Kobayashi et al., which describes enhanced osteoclast differentiation from RAW264.7 cells following PGE2 exposure and suggests EP2 and EP4 as the likely mediators^69^.

Interestingly, in our in vivo model of OA, mice with *EP4* deletion in the myeloid (osteoclast precursor cells) showed a marked improvement in most parameters measured to assess OA progression, including subchondral bone microarchitecture and osteophyte formation. However, deletion of *EP2* had little effect on improving OA progression in this model. Additionally, *EP2* deletion failed to elicit any reduction in articular cartilage degeneration or OA progression in the DMM-induced OA model (data not shown). These data suggested that EP2 might have little effect on the progression of murine OA. While *EP2* is known to be highly expressed in articular cartilage^47^, its role in OA remains unclear. *EP2* knockdown in chondrocytes has been shown to increase MMP13 production and consequently degradation of cartilage^47^. Conversely, in a separate study, the EP2 agonist butaprost suppressed proteoglycan accumulation and synthesis, aggrecan expression, and the type-II:type-I collagen ratio in human chondrocytes, exerting antianabolic effects^71^. The contradictory reports might also be explained by the lack of cross validation of data obtained on small molecules with *EP2* knockout cell and animal models.

Previous reports have shown that PGE2 can elicit a biphasic osteoclast differentiation response based on the time of exposure via its action on EP2^72^. Our in vitro study was performed at a single time point, and therefore, it is likely that we may not have captured the long-term effects of *EP2* deletion, which might have influenced the in vivo experiments. Furthermore, in our models, *EP4* deletion was a tissue-specific knockout in myeloid cells compared to global *EP2* deletion. As PGE2 has wide-ranging roles in various processes, including inflammation and repair^73^, the off-target effects of EP2 deletion may confound the results, especially after ACLT surgery.

OA is a frequent cause of pain, regardless of the complex multifactorial pathophysiology of the disease, which remains the focus for the clinical management of OA. As the cartilage is aneural, the subchondral bone, along with the other tissues of the osteochondral joint, is the primary source of nociceptive stimuli in OA^74,75^. Indeed, recent studies have shown that aberrant remodeling of subchondral bone leads to innervation of sensory nerves in patients with OA (79). Mechanistically, this phenomenon has been partly attributed to the release of NGF and Netrin-1 by osteoclasts^31,32^. Here, we show that deletion of *EP4* in osteoclasts and HL-43 both result in lower expression of Netrin-1 in the subchondral bone following ACLT-induced OA and consequently lower CGRP-positive sensory neurons. This decrease in Netrin-1 is likely to be a direct consequence of lowered osteoclast differentiation and activity, as bisphosphonates have been shown to have similar effects^31^.

Type H blood vessels are a subtype of capillaries that highly express CD31 and endomucin (Emcn) as markers. Abundant osteoprogenitors surround these vessels, with high expression of Osterix, and therefore are osteogenic in nature. In OA, type H blood vessels in the subchondral bone regulate osteoid formation, and inhibition of angiogenesis has been shown to attenuate OA progression^76^. In this study, both genetic and pharmacological inhibition of *EP4* attenuated subchondral bone angiogenesis and type H blood vessel formation. Furthermore, this effect was shown to occur via a reduction in PDGF-BB, a potent chemotactic factor secreted by osteoclasts during bone remodeling^77^. Thus, our data suggest that inhibition of PGE2/EP4-induced migration and osteoclast differentiation may prevent aberrant subchondral bone formation via a reduction in angiogenesis.

NSAIDs, including the COX-2-specific inhibitor celecoxib, are commonly used to manage OA-related pain^78–80^. However, concerns about the gastrointestinal and cardiovascular safety of COX inhibitors limit their application, and the lowest effective dose should be used for the shortest duration. Even here, we observed high cytotoxicity with celecoxib treatment in vitro (BMMs and BMSCs) and in vivo (gastrorrhagia). Nevertheless, the results from this study support the rationale of targeting the PGE2 signaling pathway in OA, but via the osteoclast EP4 receptor, to have more specific effects with lower adverse events compared to those of COX-2 inhibitors. The integral role of subchondral bone osteoclasts in OA pathogenesis is increasingly becoming evident^15,81–85^. Moreover, the suitability of targeting these subchondral bone osteoclasts for OA treatment is highlighted by trials with bisphosphonates^37,38^. However, the results from these trials have been contradictory, with some reports suggesting improved clinical outcomes^33,35,36^ and others showing no significant improvement in OA symptoms or progression^86,87^. Therefore, a novel therapeutic candidate to target subchondral bone osteoclasts could definitely be beneficial for managing OA.

In summary, our study identifies a novel mechanism by which PGE2, acting on subchondral bone osteoclasts, mediates OA pathogenesis. We provide evidence to suggest that these actions of PGE2 on osteoclasts occur via the EP4 receptor. Furthermore, targeting PGE2/EP4 in osteoclasts remarkably attenuates subchondral bone angiogenesis and sensory neuron innervation, thus reducing pain sensitivity. Finally, we also identified HL-43 as a potent EP4 antagonist, which, similar to *EP4* deletion, reduced OA progression, osteophyte formation, and pain sensitivity and improved subchondral bone microarchitecture and is therefore a novel candidate for OA treatment.

## Materials and methods

### Reagents

Celecoxib (Cat# PHR1683), safranin O solution (Cat# HT90432), fast green (Cat# F7252), hematoxylin solution (Cat# MHS1), eosin Y solution (Cat# 318906), dimethyl sulfoxide (DMSO, Cat# D8418), sodium citrate (Cat# PHR1416), hydrogen peroxide (Cat# 18304), Triton X-100 (Cat# T8787) and TRAP staining kits (Cat# 387 A) were purchased from Sigma-Aldrich (St. Louis, MO). Grapiprant (Cat# S6694), PF-04418948 (Cat# S7211), AZD6244 (Cat# S1008), JNK-IN-8 (Cat# S4901), SB203580 (Cat# S1076), GSK2141795 (Cat# S7492), neferine (Cat# S5144), IBMX (Cat# S5836) and PGE2 (Cat# S3003) were purchased from Selleck (Houston, TX). Recombinant mouse M-CSF protein (Cat# 416-ML) and recombinant mouse RANK L protein (Cat# 462-TRL) were purchased from R&D (Minnesota, USA). Matrigel (Cat# 356234) was purchased from BD Biosciences (San Jose, CA). Mountant (Cat# P36931) was purchased from Thermo Fisher Scientific (Waltham, MA). A TRACP staining kit (Cat# MK300) was purchased from TaKaRa (Dalian, China).

### Animal studies

*EP4*^*fl/fl*^ C57BL/6J mice and *EP2*^*KO*^ C57BL/6J mice were obtained from Dr. Tao P Zhong and *Gαs*^*fl/fl*^ C57BL/6J mice were a kind gift from Dr. Lee Scott Weinstein at NIDDK, National Institutes of Health. *β-arrestin1*^*KO*^ C57BL/6J mice^88^ and *β-arrestin2*^*KO*^ C57BL/6J mice were kind gifts from Dr. Gang Pei at Tongji University. *LysM-cre* C57BL/6J mice^89^ were crossed with *EP4*^*fl/fl*^ mice and *Gαs*^*fl/fl*^ mice, and the genotype of the resultant transgenic mice was determined using PCR or q-PCR of genomic DNA isolated from the mouse toe. The transgenes were genotyped using the following primer pairs: *LysM-cre*: F-5′-CCCAGAAATGCCAGATTACG-3′, and R-5′-CTTGGGCTGCCAGAATTTCTC-3′; *EP4*^*fl/fl*^: F-5′-CCCCACCCTACAGGTAAGTCG-3′, and R-5′-AGATCCAACTTCCTCATCGGTA-3′; *Gαs*^*fl/f*l^: F-5′-TTCGGCTCGTCCCCTTAGTTG-3′, and R-5′-AACAAATCGCACACCCCAGTGAGG-3′; *EP2*: F-5′-TGCTCATGCTCTTCGCTATG-3′, and R-5′-CGTACTCCCCGTAGTTGAGC-3′; *β-arrestin1*: F-5′-CCTAGTGCTGGGATTACAAG-3′, and R-5′-CATAGCCTGAAGAACGAGAT-3′; *β-arrestin2*: F-5′-GCTAAAGCGCATGCTCCAGA-3′, and R-5′-ACAGGGTCCACTTTGTCCA-3′.

### ACLT surgery

The ACL was transected to induce OA in 10-week-old male C57BL/6 J mice as previously described^15^. The mice were subsequently euthanized at either 14 (2 weeks) or 56 days (8 weeks) after surgery (*n* = 6 per group).

We also purchased 10-week-old male WT C57BL/6 J mice from the National Rodent Laboratory Animal Resources (Shanghai, China) and performed sham or ACLT surgery. The mice were then treated every day for 2 weeks or 8 weeks after surgery with 30 mg per kg body weight celecoxib, and HL-43 was resuspended in 0.5% sodium carboxymethylcellulose (Sigma St. Louis, MO) (n = 4-21 per group). The sham and ACLT vehicle control mice were treated with 0.5% sodium carboxymethylcellulose, and all treatments were administered by gavage. At the end of the procedure, the mice were euthanized, and knee joints were collected for histological analyses. The OARSI histopathology grading system was used to evaluate the sections as previously described^90^. Mice were caged in a laminar airflow cabinet under specific pathogen-free conditions. They were kept at 22 °C, fed sterilized water and food (Xie Tong Biomedical Engineering Company, Jiangsu, China; Cat# 1010084), and kept on a 12 h light/dark cycle. We maintained all mice in the animal facility of East China Normal University (Shanghai, China). The experimental protocol was approved by the Institutional Animal Care and Use Committee of East China Normal University. The assigned approval number of this study was m + R20190301.

### Mechanical nociception hyperalgesia test

The sensitivity to punctate static mechanical stimuli was assessed in the mouse hind paw by von Frey nylon filaments. Mice were placed in cages for 30 min prior to the test. Von Frey filaments (Ugo Basile, Varese, Italy) were applied to the midplantar surface of hind paws under the mesh cage. We recorded the minimal force to elicit a 50% positive response as the paw withdrawal threshold. The investigators were blinded to the study groups and performed all tests.

### Thermal nociception hyperalgesia test

Thermal nociceptive hyperalgesia was measured by the plantar test using an Ugo-Basile 37370 (Comerio, Italy). The mice were placed on a transparent glass plate for 30 min prior to the test, thermal nociception hyperalgesia of the ipsilateral hind paws was assessed three times, and there was a 30-minute interval between each trial. We recorded the time(s) from irradiation to mouse hind paw withdrawal as the thermal withdrawal latency. The investigators were blinded to the study groups and performed all tests.

### Primary osteoclast culture, migration and differentiation assays

Bone marrow macrophages (BMMs) were isolated from 8-week-old male C57BL/6 J mouse femurs and tibiae as previously described^89^. Briefly, cells were isolated by flushing bone marrow of femur and tibia and cultured in α-MEM (Gibco) with 10% serum (Gibco), 1% penicillin-streptomycin (HyClone) and 10 ng·mL^−1^ M-CSF (R&D) to generate BMMs. For differentiation, BMMs were seeded in 96-well culture plates (1 × 10^4^ cells per well) and induced with 10 ng·mL^−1^ M-CSF and 50 ng·mL^−1^ RANKL (R&D) for 5-6 days. When BMMs differentiated into mature osteoclasts, a TRAP staining kit (TaKaRa) was used to stain mature osteoclasts according to the manufacturer′s instructions, and TRAP-positive osteoclasts with 5 or more nuclei were counted. For BMM migration, BMMs (1 × 10^4^ cells per well) were seeded in the upper chamber of the Transwell inserts with or without inhibitors and antagonists. PGE2 was added in lower chamber. The culture medium was the same between the upper chamber and lower chamber in the Transwell. After 18 h, the cells were fixed with 4% PFA and stained with crystal violet. The cells on the bottom of the Transwell insert were used to assess migration. At least five fields of view per insert were photographed, and migrated cells were counted by Image-Pro Plus 6.0 software (Media Cybernetics).

### Immunohistochemical staining and immunofluorescence staining

Mouse knee joint tissue was fixed in 4% paraformaldehyde for 48 h and decalcified in 10% EDTA for 2 weeks. The tissues were embedded in paraffin or optimal cutting temperature (OCT) and sectioned at a thickness of 6 µm for immunohistochemical staining or 25 µm for immunofluorescence staining. A standard protocol was followed for immunohistochemical staining^91^. Briefly, tissue sections were soaked in xylene for 8 min and replaced with fresh xylene for 5 min. Subsequently, the tissue slices were soaked in a series of ethanol solutions (100%, 95%, 85% and 75%) to deplete xylene. Citrate buffer (Sigma) was used to perform antigen retrieval, and 3% hydrogen peroxide (Sigma) was used to reduce endogenous peroxidase activity. The tissue sections were permeabilized with 0.1% Triton X-100 (Sigma). Tissue sections were blocked in 2% horse serum to reduce nonspecific staining and then incubated with the indicated antibodies. Anti-MMP13 (Cat# ab219620, 1:200 dilution) was purchased from Abcam (Cambridge, MA). An immunohistochemistry Application Solutions Kit (#13079, CST) was used, and a Leica microscope (Leica, DM4000b) was used to obtain images. Cells with positive signals were counted in the tibia subchondral bone and cartilage specimens, with three sequential specimens. A negative control in which the primary antibody was replaced by 2% goat serum was performed to exclude nonspecific signals. A standard protocol was followed for immunofluorescence staining^91^. Briefly, frozen tissue sections were dried for 15 min at room temperature and fixed in 4% paraformaldehyde for 10 min. Triton X-100 (0.1%, Sigma) was used to permeate tissue slices. Tissue sections were blocked in 2% horse serum to reduce nonspecific staining and were subsequently incubated with the indicated antibodies. Anti-EP2 (Cat# ab167171, 1:200 dilution), anti-Collagen X (Cat# ab182563, 1:1 000 dilution), anti-Netrin-1 (Cat# ab39370, 1:100 dilution), anti-PDGF-BB (Cat# ab23914, 1:100 dilution), anti-CGRP (Cat# ab81887, 1:200 dilution), anti-Osterix (Cat# ab22552, 1:100 dilution), anti-CD31 (Cat# ab24590, 1:100 dilution) and anti-CD115 (Cat# ab272049, 1:200 dilution) were purchased from Abcam (Cambridge, MA). Anti-EP4 (Cat# sc-55596, 1:100 dilution), anti-EMCN (Cat# sc-65495, 1:50 dilution) and anti-RANK (Cat# sc-374360, 1:100 dilution) were obtained from Santa Cruz (Texas, USA). Anti-TRAP (Cat# 32694, 1:200 dilution) was purchased from SAB (Maryland, USA) and used for TRAP IHF staining and TRAP and EP4 IHF double staining. Anti-TRAP (Cat# NB300-555, 1:200 dilution) was purchased from Novus (Colorado, USA) and used for TRAP and EP2 IHF double staining. Tissue sections were incubated with appropriate fluorescently labeled secondary antibody for 1 h, and images were obtained with a Leica microscope (Leica, DM4000b) and two-photon laser confocal microscope (Leica, TCS SP8). Cells with positive signals were counted in the tibia subchondral bone and cartilage specimens, with three sequential specimens. Similar to immunohistochemistry, a no-primary negative control was added to each batch of slides to preclude nonspecific signals, and a Vector® TrueVIEW® Autofluorescence Quenching Kit (Vector Labs, Cat#: SP-8400-15) was also used to minimize nonspecific IHF signals.

### Western blotting analysis

Western blotting analysis was performed as previously described^92^. Briefly, mouse knee joint tissues and human knee joint subchondral bone tissues were ground in liquid nitrogen and lysed in modified radioimmunoprecipitation assay buffer, and osteoclasts were also lysed with the same buffer. A BCA Protein Assay Kit (Thermo Fisher Scientific) was used to determine the protein concentration. The same amounts of protein were resolved by SDS-PAGE electrophoresis and transferred to nitrocellulose membranes (Millipore). Then, 5% BSA (Sigma) was used to block membranes. Phosphate buffer solution was used to wash membranes, and specific antibodies were immunoblotted with membranes. The detailed information of the antibodies used is as follows: anti-EP2 (Abcam Cat# ab167171, 1:1 000 dilution), anti-EP4 (Santa Cruz Cat# sc-55596, 1:100 dilution), anti-PDGF-BB (Abcam Cat# ab23914, 1:1 000 dilution), anti-Netrin-1 (Abcam Cat# ab126729, 1:1 000 dilution), anti-p65 (CST Cat# 8242, 1:1 000 dilution), anti-p-p65 (CST Cat# 3033, 1:1 000 dilution), anti-AKT (CST Cat# 4685 S, 1:1 000 dilution), anti-p-ATK (CST Cat# 13038 S, 1:1 000 dilution), anti-ERK (CST Cat# 4695, 1:1 000 dilution), anti-p-ERK (CST Cat# 4370, 1:1 000 dilution), anti-p38 (CST Cat# 8690, 1:1 000 dilution), anti-p-p38 (CST Cat# 4511, 1:1 000 dilution), anti-JNK (CST Cat# 9252, 1:1 000 dilution), anti-p-JNK (CST Cat# 4668, 1:1 000 dilution) and anti-GAPDH (Abcam Cat# ab9485, 1:10 000 dilution). Following overnight incubation, the membranes were washed with TBST and incubated with secondary antibodies (LI-COR Biosciences, Cat# 926-32211, 1: 10 000 dilution) without light for 1 h. The membranes were then washed with TBST, and an Odyssey Infrared Imaging System was used to obtain images.

### Quantitative real-time PCR

We used RNAiso Plus (TaKaRa, Dalian, China) to extract RNA and then converted it to cDNA by a Prime Script RT Reagent Kit (TaKaRa, Dalian, China). A SYBR kit (TaKaRa, Dalian, China) and 96-well Thermal iCycler (Bio-Rad, Hercules, CA) were used to perform qRT-PCR. Detailed information on the primers is listed as follows.

*EP1*: F-5′-GGGCTTAACCTGAGCCTAGC-3′, and R-5′-GTGATGTGCCATTATCGCCTG-3′; *EP2*: F-5′-GTAACGGAATTGGTGCTCACT-3′, and R-5′-TGAAAGCGAAATAGGTACACGC-3′; *EP3*: F-5′-CCGGAGCACTCTGCTGAAG-3′, and R-5′-CCCCACTAAGTCGGTGAGC-3′; *EP4*: F-5′-GTGCGGAGATCCAGATGGTC-3′, and R-5′-TCACCACGTTTGGCTGATATAAC-3′; *Trap*: F-5′-CACTCCCACCCTGAGATTTGT-3′, and R-5′-CCCCAGAGACATGATGAAGTCA-3′; *Pdgf-bb*: F-5′-CATCCGCTCCTTTGATGATCTT-3′, and R-5′-GTGCTCGGGTCATGTTCAAGT-3′; *Vegf-A*: F-5′-CTGCCGTCCGATTGAGACC-3′, and R-5′-CCCCTCCTTGTACCACTGTC-3′; *Angiogenin*: F-5′-CCAGGCCCGTTGTTCTTGAT-3′, and R-5′-GGAAGGGAGACTTGCTCATTC-3′; *Slit3*: F-5′-TGCCCCACCAAGTGTACCT-3′, and R-5′-GGCCAGCGAAGTCCATTTTG-3′; *Ngf*: F-5′-AGACTCCACTCACCCCGTG-3′, and R-5′-GGCTGTGGTCTTATCTCCAAC-3′; *Netrin-1*: F-5′-CGACCTCAATAACCCGCACAA-3′, and R-5′-GCGTGGAATAGAACTGGAAGG-3′. *Gapdh*: F-5′-TGGCCTTCCGTGTTCCTAC-3′, and R-5′-GAGTTGCTGTTGAAGTCGCA-3′;

### Cell toxicity assay

BMMs (stimulated with 10 ng·mL^−1^ M-CSF) and BMSCs were seeded in 96-well culture plates (5 × 10^3^–8 × 10^3^ cells per well for BMMs and 3 × 10^3^–5 × 10^3^ cells per well for BMSCs) and allowed to adhere overnight. Subsequently, the cells were treated for 48 h with different drug concentrations. Cell viability was measured by a cell proliferation assay kit (Promega, Madison, WI) according to the manufacturer’s instructions. The IC50 values were calculated by GraphPad Prism 8.0 (log [inhibitor] vs. normalized response).

### MicroCT

Then, 4% paraformaldehyde was used to fix knee joints, which were dissected from mice overnight, and high-resolution µCT (Skyscan-1272; Bruker microCT, Belgium) was used to scan the tissues. The scanner operated at a voltage of 60 kV, 166 µA, and a resolution of 7 µm per pixel. Two-dimensional images were reconstructed by Skyscan NRecon software (Bruker), and CTAnalyser software (Bruker) was used to analyze the images. CTVox software (Bruker) was used to analyze the parameters of the trabecular bone. Three-dimensional histomorphometric analyses were conducted on the sagittal images of the tibial subchondral bone. The entire subchondral bone medial compartment, consisting of fifteen consecutive images from the medial tibial plateau, was analyzed. The three-dimensional structural parameters analyzed included BV/TV, Tb.Sp, and Tb.Pf.

### HUVEC tube formation

Matrigel (BD Biosciences) was thawed in a refrigerator at 4 °C overnight, plated in 48-well culture plates and polymerized in a cell incubator. Subsequently, HUVECs obtained from ATCC (Manassas, VA) (2 × 10^4^ cells per well) were seeded on polymerized Matrigel. Monocytes were harvested from 8-week-old WT, *Ep4*^*fl/fl*^, and *Ep4*^*LysM*^ male mice by flushing the bone marrow of femurs and tibia and differentiated into osteoclast precursor cells over 3 days using media with 60 ng·mL^−1^ RANKL, 10 ng·mL^−1^ M-CSF, and 100 nmol·L^−1^ PGE2. Conditioned medium from osteoclast precursor cells was harvested and concentrated in PES protein concentrators (Thermo Fisher Scientific) and then used to induce tube formation. Images were obtained by microscopy, and GraphPad Prism 8.0 was used to measure the cumulative branching length and number of tube nodes.

### Phosphoproteome antibody array

BMMs isolated from the *EP4*^*LysM*^ and *EP4*^*fl/fl*^ mice were stimulated with 10 ng·mL^−1^ M-CSF, 50 ng·mL^−1^ RANKL, and 100 nmol·L^−1^ PGE2 for 3 h. The cell lysates were collected and subjected to a phosphoproteome antibody array containing 157 phosphoprotein-targeted antibodies (Full Moon Biosystems, Phospho Explorer CSP100_Plus). The levels of the individual proteins were normalized to that of β-actin (included in the array as an internal control).

### Human joint subchondral bone samples

Human joint tissues with subchondral bone undergoing total knee arthroplasty were obtained from Shanghai Sixth People’s Hospital, and the subchondral bone from the polydactyly patient undergoing resection was obtained as a control, with the approval of the Human Ethics Committee (2021-048). We scraped the cartilage off cleanly and then used pieces of subchondral bone from the different samples for protein extraction and western blotting.

### Statistical analysis

All data are presented as the mean ± S.D. One-way ANOVA followed by Tukey’s t tests, two-way ANOVA followed by Tukey’s *t* tests or two-tailed paired Student’s *t* tests were used to compare the means among groups. GraphPad Prism 8.0 was used to perform statistical tests.

## Supplementary information

Supplementary figure

Supplemental Figure

## Data Availability

All data are available upon reasonable request from the corresponding author with the publication.

## References

[1] Collaborators, G. B. D. R. F. Global, regional, and national comparative risk assessment of 84 behavioural, environmental and occupational, and metabolic risks or clusters of risks for 195 countries and territories, 1990–2017: a systematic analysis for the Global Burden of Disease Study 2017. *Lancet* **392**, 1923–1994 (2018). 30496105 10.1016/S0140-6736(18)32225-6 PMC6227755

[2] Peat, G. & Thomas, M. J. Osteoarthritis year in review 2020: epidemiology & therapy. *Osteoarthr. Cartil*. **29**, 180–189 (2021). 10.1016/j.joca.2020.10.007 33242603

[3] Liu, Q., Wang, S., Lin, J. & Zhang, Y. The burden for knee osteoarthritis among Chinese elderly: estimates from a nationally representative study. *Osteoarthr. Cartil.* **26**, 1636–1642 (2018). 10.1016/j.joca.2018.07.019 30130589

[4] Robinson, W. H. et al. Low-grade inflammation as a key mediator of the pathogenesis of osteoarthritis. *Nat. Rev. Rheumatol.* **12**, 580–592 (2016). 27539668 10.1038/nrrheum.2016.136 PMC5500215

[5] Madry, H. The subchondral bone: a new frontier in articular cartilage repair. *Knee Surg. Sports Traumatol. Arthrosc.* **18**, 417–418 (2010). 20127311 10.1007/s00167-010-1071-y

[6] Goldring, S. R. Alterations in periarticular bone and cross talk between subchondral bone and articular cartilage in osteoarthritis. *Ther. Adv. Musculoskelet. Dis.* **4**, 249–258 (2012). 22859924 10.1177/1759720X12437353 PMC3403248

[7] Kloppenburg, M. & Berenbaum, F. Osteoarthritis year in review 2019: epidemiology and therapy. *Osteoarthr. Cartil.* **28**, 242–248 (2020). 10.1016/j.joca.2020.01.002 31945457

[8] Nelson, A. E. Osteoarthritis year in review 2017: clinical. *Osteoarthr. Cartil.* **26**, 319–325 (2018). 10.1016/j.joca.2017.11.014 PMC5835411 29229563

[9] Farnaghi, S., Crawford, R., Xiao, Y. & Prasadam, I. Cholesterol metabolism in pathogenesis of osteoarthritis disease. *Int. J. Rheum. Dis.* **20**, 131–140 (2017). 28378420 10.1111/1756-185X.13061

[10] Choi, W. S. et al. The CH25H-CYP7B1-RORalpha axis of cholesterol metabolism regulates osteoarthritis. *Nature* **566**, 254–258 (2019). 30728500 10.1038/s41586-019-0920-1

[11] Chang, S. H. et al. Excessive mechanical loading promotes osteoarthritis through the gremlin-1-NF-kappaB pathway. *Nat. Commun.* **10**, 1442 (2019). 30926814 10.1038/s41467-019-09491-5 PMC6441020

[12] Jimenez, G., Cobo-Molinos, J., Antich, C. & Lopez-Ruiz, E. Osteoarthritis: trauma vs disease. *Adv. Exp. Med. Biol.* **1059**, 63–83 (2018). 29736569 10.1007/978-3-319-76735-2_3

[13] Palazzo, C., Nguyen, C., Lefevre-Colau, M. M., Rannou, F. & Poiraudeau, S. Risk factors and burden of osteoarthritis. *Ann. Phys. Rehab. Med.* **59**, 134–138 (2016). 10.1016/j.rehab.2016.01.006 26904959

[14] Cui, Z. et al. Halofuginone attenuates osteoarthritis by inhibition of TGF-beta activity and H-type vessel formation in subchondral bone. *Ann. Rheum. Dis.* **75**, 1714–1721 (2016). 26470720 10.1136/annrheumdis-2015-207923 PMC5013081

[15] Zhen, G. et al. Inhibition of TGF-beta signaling in mesenchymal stem cells of subchondral bone attenuates osteoarthritis. *Nat. Med.* **19**, 704–712 (2013). 23685840 10.1038/nm.3143 PMC3676689

[16] Su, W. et al. Angiogenesis stimulated by elevated PDGF-BB in subchondral bone contributes to osteoarthritis development. *JCI Insight* **5**, e135446 (2020). 10.1172/jci.insight.135446 PMC7205438 32208385

[17] Lin, C. et al. Activation of mTORC1 in subchondral bone preosteoblasts promotes osteoarthritis by stimulating bone sclerosis and secretion of CXCL12. *Bone Res.* **7**, 5 (2019). 30792936 10.1038/s41413-018-0041-8 PMC6381187

[18] Lories, R. J. & Luyten, F. P. The bone-cartilage unit in osteoarthritis. *Nat. Rev. Rheumatol.* **7**, 43–49 (2011). 21135881 10.1038/nrrheum.2010.197

[19] Cinque, M. E., Dornan, G. J., Chahla, J., Moatshe, G. & LaPrade, R. F. High rates of osteoarthritis develop after anterior cruciate ligament surgery: an analysis of 4108 patients. *Am. J. Sports Med.* **46**, 2011–2019 (2018). 28982255 10.1177/0363546517730072

[20] Thijssen, E., van Caam, A. & van der Kraan, P. M. Obesity and osteoarthritis, more than just wear and tear: pivotal roles for inflamed adipose tissue and dyslipidaemia in obesity-induced osteoarthritis. *Rheumatology* **54**, 588–600 (2015). 25504962 10.1093/rheumatology/keu464

[21] de Zwart, A. H. et al. Factors associated with upper leg muscle strength in knee osteoarthritis: a scoping review. *J. Rehab. Med.* **50**, 140–150 (2018). 10.2340/16501977-2284 29186637

[22] Li, G. et al. Influence of age and gender on microarchitecture and bone remodeling in subchondral bone of the osteoarthritic femoral head. *Bone* **77**, 91–97 (2015). 25892484 10.1016/j.bone.2015.04.019

[23] Roemer, F. W. et al. Change in MRI-detected subchondral bone marrow lesions is associated with cartilage loss: the MOST Study. A longitudinal multicentre study of knee osteoarthritis. *Ann. Rheum. Dis.* **68**, 1461–1465 (2009). 18829615 10.1136/ard.2008.096834 PMC2905622

[24] Hunter, D. J. et al. Increase in bone marrow lesions associated with cartilage loss: a longitudinal magnetic resonance imaging study of knee osteoarthritis. *Arthritis Rheumat.* **54**, 1529–1535 (2006). 16646037 10.1002/art.21789

[25] Raynauld, J. P. et al. Correlation between bone lesion changes and cartilage volume loss in patients with osteoarthritis of the knee as assessed by quantitative magnetic resonance imaging over a 24-month period. *Ann. Rheum. Dis.* **67**, 683–688 (2008). 17728333 10.1136/ard.2007.073023

[26] Raggatt, L. J. & Partridge, N. C. Cellular and molecular mechanisms of bone remodeling. *J. Biol. Chem.* **285**, 25103–25108 (2010). 20501658 10.1074/jbc.R109.041087 PMC2919071

[27] Lacourt, M. et al. Relationship between cartilage and subchondral bone lesions in repetitive impact trauma-induced equine osteoarthritis. *Osteoarthr. Cartil.* **20**, 572–583 (2012). 10.1016/j.joca.2012.02.004 22343573

[28] Zhen, G. & Cao, X. Targeting TGFbeta signaling in subchondral bone and articular cartilage homeostasis. *Trends Pharmacol. Sci.* **35**, 227–236 (2014). 24745631 10.1016/j.tips.2014.03.005 PMC4058854

[29] Peng, Y., Wu, S., Li, Y. & Crane, J. L. Type H blood vessels in bone modeling and remodeling. *Theranostics* **10**, 426–436 (2020). 31903130 10.7150/thno.34126 PMC6929606

[30] Hu, W., Chen, Y., Dou, C. & Dong, S. Microenvironment in subchondral bone: predominant regulator for the treatment of osteoarthritis. *Ann. Rheum. Dis.* (2020). 10.1136/annrheumdis-2020-218089 PMC7958096 33158879

[31] Zhu, S. et al. Subchondral bone osteoclasts induce sensory innervation and osteoarthritis pain. *J. Clin. Investig.* **129**, 1076–1093 (2019). 30530994 10.1172/JCI121561 PMC6391093

[32] Nwosu, L. N., Mapp, P. I., Chapman, V. & Walsh, D. A. Blocking the tropomyosin receptor kinase A (TrkA) receptor inhibits pain behaviour in two rat models of osteoarthritis. *Ann. Rheum. Dis.* **75**, 1246–1254 (2016). 26286016 10.1136/annrheumdis-2014-207203 PMC4893148

[33] Laslett, L. L. et al. Zoledronic acid reduces knee pain and bone marrow lesions over 1 year: a randomised controlled trial. *Ann. Rheum. Dis.* **71**, 1322–1328 (2012). 22355040 10.1136/annrheumdis-2011-200970

[34] Ballal, P. et al. The relation of oral bisphosphonates to bone marrow lesion volume among women with osteoarthritis. *Osteoarthr. Cartil.* **28**, 1325–1329 (2020). 10.1016/j.joca.2020.07.006 PMC7530037 32768598

[35] Varenna, M., Zucchi, F., Failoni, S., Becciolini, A. & Berruto, M. Intravenous neridronate in the treatment of acute painful knee osteoarthritis: a randomized controlled study. *Rheumatology* **54**, 1826–1832 (2015). 25998450 10.1093/rheumatology/kev123

[36] Rossini, M. et al. Effects of intra-articular clodronate in the treatment of knee osteoarthritis: results of a double-blind, randomized placebo-controlled trial. *Rheumatol. Int.* **35**, 255–263 (2015). 25080876 10.1007/s00296-014-3100-5

[37] Vaysbrot, E. E., Osani, M. C., Musetti, M. C., McAlindon, T. E. & Bannuru, R. R. Are bisphosphonates efficacious in knee osteoarthritis? A meta-analysis of randomized controlled trials. *Osteoarthr. Cartil.* **26**, 154–164 (2018). 10.1016/j.joca.2017.11.013 29222056

[38] Eriksen, E. F., Shabestari, M., Ghouri, A. & Conaghan, P. G. Bisphosphonates as a treatment modality in osteoarthritis. *Bone* **143**, 115352 (2021). 32247817 10.1016/j.bone.2020.115352

[39] Zhang, Y. & Daaka, Y. PGE2 promotes angiogenesis through EP4 and PKA Cgamma pathway. *Blood* **118**, 5355–5364 (2011). 21926356 10.1182/blood-2011-04-350587 PMC3217416

[40] Lu, W. et al. Reprogramming immunosuppressive myeloid cells facilitates immunotherapy for colorectal cancer. *EMBO Mol. Med.* **13**, e12798 (2020). 10.15252/emmm.202012798 PMC7799360 33283987

[41] Ni, S. et al. Sensory innervation in porous endplates by Netrin-1 from osteoclasts mediates PGE2-induced spinal hypersensitivity in mice. *Nat. Commun.* **10**, 5643 (2019). 31822662 10.1038/s41467-019-13476-9 PMC6904550

[42] Nakanishi, M. & Rosenberg, D. W. Multifaceted roles of PGE2 in inflammation and cancer. *Semin. Immunopathol.* **35**, 123–137 (2013). 22996682 10.1007/s00281-012-0342-8 PMC3568185

[43] Jin, J. et al. Prostaglandin E2 regulates renal function in C57/BL6 mouse with 5/6 nephrectomy. *Life Sci.* **174**, 68–76 (2017). 28263803 10.1016/j.lfs.2017.03.001

[44] Tu, M. et al. Inhibition of cyclooxygenase-2 activity in subchondral bone modifies a subtype of osteoarthritis. *Bone Res.* **7**, 29 (2019). 31666999 10.1038/s41413-019-0071-x PMC6804921

[45] Amin, A. R. et al. Superinduction of cyclooxygenase-2 activity in human osteoarthritis-affected cartilage. Influence of nitric oxide. *J. Clin. Investig.* **99**, 1231–1237 (1997). 9077531 10.1172/JCI119280 PMC507937

[46] Sugimoto, Y. & Narumiya, S. Prostaglandin E receptors. *J. Biol. Chem.* **282**, 11613–11617 (2007). 17329241 10.1074/jbc.R600038200

[47] Sato, T. et al. Prostaglandin EP2 receptor signalling inhibits the expression of matrix metalloproteinase 13 in human osteoarthritic chondrocytes. *Ann. Rheum. Dis.* **70**, 221–226 (2011). 20870807 10.1136/ard.2009.118620

[48] Weinreb, M. et al. Expression of the prostaglandin E(2) (PGE(2)) receptor subtype EP(4) and its regulation by PGE(2) in osteoblastic cell lines and adult rat. *Bone Tissue Bone* **28**, 275–281 (2001). 11248657 10.1016/s8756-3282(00)00447-6

[49] Yoshida, K. et al. Stimulation of bone formation and prevention of bone loss by prostaglandin E EP4 receptor activation. *Proc. Natl. Acad. Sci. USA* **99**, 4580–4585 (2002). 11917107 10.1073/pnas.062053399 PMC123690

[50] Attur, M. et al. Prostaglandin E2 exerts catabolic effects in osteoarthritis cartilage: evidence for signaling via the EP4 receptor. *J. Immunol.* **181**, 5082–5088 (2008). 18802112 10.4049/jimmunol.181.7.5082

[51] Nishitani, K. et al. PGE2 inhibits MMP expression by suppressing MKK4-JNK MAP kinase-c-JUN pathway via EP4 in human articular chondrocytes. *J. Cell. Biochem.* **109**, 425–433 (2010). 19998410 10.1002/jcb.22421

[52] Ashraf, S. et al. Augmented pain behavioural responses to intra-articular injection of nerve growth factor in two animal models of osteoarthritis. *Ann. Rheum. Dis.* **73**, 1710–1718 (2014). 23852764 10.1136/annrheumdis-2013-203416 PMC4145450

[53] Hunter, D. J. & Bierma-Zeinstra, S. Osteoarthritis. *Lancet* **393**, 1745–1759 (2019). 31034380 10.1016/S0140-6736(19)30417-9

[54] Tombran-Tink, J. & Barnstable, C. J. Osteoblasts and osteoclasts express PEDF, VEGF-A isoforms, and VEGF receptors: possible mediators of angiogenesis and matrix remodeling in the bone. *Biochem. Biophys. Res. Commun.* **316**, 573–579 (2004). 15020256 10.1016/j.bbrc.2004.02.076

[55] Kim, B. J. et al. Osteoclast-secreted SLIT3 coordinates bone resorption and formation. *J. Clin. Investig.* **128**, 1429–1441 (2018). 29504949 10.1172/JCI91086 PMC5873876

[56] Liu, X. et al. Osteoclasts protect bone blood vessels against senescence through the angiogenin/plexin-B2 axis. *Nat. Commun.* **12**, 1832 (2021). 33758201 10.1038/s41467-021-22131-1 PMC7987975

[57] Yang, J. J. et al. Discovery and characterization of 1H-1,2,3-triazole derivatives as novel prostanoid EP4 receptor antagonists for cancer immunotherapy. *J. Med. Chem.* **63**, 569–590 (2020). 31855426 10.1021/acs.jmedchem.9b01269

[58] Rane, M. A., Gitin, A., Fiedler, B., Fiedler, L. & Hennekens, C. H. Risks of cardiovascular disease and beyond in prescription of nonsteroidal anti-inflammatory drugs. *J. Cardiovasc. Pharmacol. Ther.* **25**, 3–6 (2020). 31466474 10.1177/1074248419871902

[59] Walker, C. & Biasucci, L. M. Cardiovascular safety of non-steroidal anti-inflammatory drugs revisited. *Postgrad. Med.* **130**, 55–71 (2018). 29202670 10.1080/00325481.2018.1412799

[60] Hawkey, C. J. COX-1 and COX-2 inhibitors. *Best. Pract. Res. Clin. Gastroenterol.* **15**, 801–820 (2001). 11566042 10.1053/bega.2001.0236

[61] Bjarnason, I. Gastrointestinal safety of NSAIDs and over-the-counter analgesics. *Int. J. Clin. Pract. Supplement*, 37-42, (2013). 10.1111/ijcp.12048 23163547

[62] Gao, M. et al. Disruption of prostaglandin E2 receptor EP4 impairs urinary concentration via decreasing aquaporin 2 in renal collecting ducts. *Proc. Natl Acad. Sci. USA* **112**, 8397–8402 (2015). 26100911 10.1073/pnas.1509565112 PMC4500247

[63] Johnston, J. E. The best medicine sometimes can’t be bought. *J. Miss. State Med. Assoc.* **33**, 97–98 (1992). 1564728

[64] Yokoyama, U., Iwatsubo, K., Umemura, M., Fujita, T. & Ishikawa, Y. The prostanoid EP4 receptor and its signaling pathway. *Pharmacol. Rev.* **65**, 1010–1052 (2013). 23776144 10.1124/pr.112.007195

[65] Li, D. Z., Zhang, Q. X., Dong, X. X., Li, H. D. & Ma, X. Treatment with hydrogen molecules prevents RANKL-induced osteoclast differentiation associated with inhibition of ROS formation and inactivation of MAPK, AKT and NF-kappa B pathways in murine RAW264.7 cells. *J. Bone Miner. Metab.* **32**, 494–504 (2014). 24196871 10.1007/s00774-013-0530-1

[66] Mendoza, M. C., Er, E. E. & Blenis, J. The Ras-ERK and PI3K-mTOR pathways: cross-talk and compensation. *Trends Biochem. Sci.* **36**, 320–328 (2011). 21531565 10.1016/j.tibs.2011.03.006 PMC3112285

[67] Kalbasi Anaraki, P. et al. Urokinase receptor mediates osteoclastogenesis via M-CSF release from osteoblasts and the c-Fms/PI3K/Akt/NF-kappaB pathway in osteoclasts. *J. Bone Miner. Res.* **30**, 379–388 (2015). 25196912 10.1002/jbmr.2350

[68] Hayami, T. et al. The role of subchondral bone remodeling in osteoarthritis: reduction of cartilage degeneration and prevention of osteophyte formation by alendronate in the rat anterior cruciate ligament transection model. *Arthritis Rheumat.* **50**, 1193–1206 (2004). 15077302 10.1002/art.20124

[69] Kobayashi, Y. et al. Prostaglandin E2 enhances osteoclastic differentiation of precursor cells through protein kinase A-dependent phosphorylation of TAK1. *J. Biol. Chem.* **280**, 11395–11403 (2005). 15647289 10.1074/jbc.M411189200

[70] Mano, M. et al. Prostaglandin E2 directly inhibits bone-resorbing activity of isolated mature osteoclasts mainly through the EP4 receptor. *Calcif. Tissue Int.* **67**, 85–92 (2000). 10908419 10.1007/s00223001102

[71] Li, X. et al. Prostaglandin E2 and its cognate EP receptors control human adult articular cartilage homeostasis and are linked to the pathophysiology of osteoarthritis. *Arthritis Rheumat.* **60**, 513–523 (2009). 19180509 10.1002/art.24258 PMC2659545

[72] Ono, K. et al. Biphasic effect of prostaglandin E2 on osteoclast formation in spleen cell cultures: role of the EP2 receptor. *J. Bone Miner. Res.* **20**, 23–29 (2005). 15619666 10.1080/14041040510033842

[73] Gilman, K. E. & Limesand, K. H. The complex role of prostaglandin E2-EP receptor signaling in wound healing. *Am. J. Physiol. Regulatory, Integr. Comp. Physiol.* **320**, R287–R296 (2021). 10.1152/ajpregu.00185.2020 PMC7988772 33296281

[74] Dieppe, P. A. & Lohmander, L. S. Pathogenesis and management of pain in osteoarthritis. *Lancet* **365**, 965–973 (2005). 15766999 10.1016/S0140-6736(05)71086-2

[75] Walsh, D. A. et al. Angiogenesis and nerve growth factor at the osteochondral junction in rheumatoid arthritis and osteoarthritis. *Rheumatology* **49**, 1852–1861 (2010). 20581375 10.1093/rheumatology/keq188 PMC2936950

[76] Lu, J. et al. Positive-feedback regulation of subchondral H-type vessel formation by chondrocyte promotes osteoarthritis development in mice. *J. Bone Miner. Res.* **33**, 909–920 (2018). 29329496 10.1002/jbmr.3388

[77] Xie, H. et al. PDGF-BB secreted by preosteoclasts induces angiogenesis during coupling with osteogenesis. *Nat. Med.* **20**, 1270–1278 (2014). 25282358 10.1038/nm.3668 PMC4224644

[78] Puljak, L. et al. Celecoxib for osteoarthritis. *Cochrane Database Syst. Rev.* **5**, CD009865 (2017). 28530031 10.1002/14651858.CD009865.pub2 PMC6481745

[79] Angeli, F., Trapasso, M., Signorotti, S., Verdecchia, P. & Reboldi, G. Amlodipine and celecoxib for treatment of hypertension and osteoarthritis pain. *Expert Rev. Clin. Pharmacol.* **11**, 1073–1084 (2018). 30362840 10.1080/17512433.2018.1540299

[80] Clemett, D. & Goa, K. L. Celecoxib: a review of its use in osteoarthritis, rheumatoid arthritis and acute pain. *Drugs* **59**, 957–980 (2000). 10804043 10.2165/00003495-200059040-00017

[81] Li, G. et al. Subchondral bone in osteoarthritis: insight into risk factors and microstructural changes. *Arthritis Res. Ther.* **15**, 223 (2013). 24321104 10.1186/ar4405 PMC4061721

[82] Aaron, R. K., Racine, J. R., Voisinet, A., Evangelista, P. & Dyke, J. P. Subchondral bone circulation in osteoarthritis of the human knee. *Osteoarthr. Cartil.* **26**, 940–944 (2018). 10.1016/j.joca.2018.04.003 29723635

[83] Burr, D. B. & Gallant, M. A. Bone remodelling in osteoarthritis. *Nat. Rev. Rheumatol.* **8**, 665–673 (2012). 22868925 10.1038/nrrheum.2012.130

[84] Mansell, J. P., Collins, C. & Bailey, A. J. Bone, not cartilage, should be the major focus in osteoarthritis. *Nat. Clin. Pract. Rheumatol.* **3**, 306–307 (2007). 17538562 10.1038/ncprheum0505

[85] Martel-Pelletier, J. et al. Osteoarthritis. *Nat. Rev. Dis. Prim.* **2**, 16072 (2016). 27734845 10.1038/nrdp.2016.72

[86] Spector, T. D. et al. Effect of risedronate on joint structure and symptoms of knee osteoarthritis: results of the BRISK randomized, controlled trial [ISRCTN01928173]. *Arthritis Res. Ther.* **7**, R625–R633 (2005). 15899049 10.1186/ar1716 PMC1174954

[87] Bingham, C. O. 3rd et al. Risedronate decreases biochemical markers of cartilage degradation but does not decrease symptoms or slow radiographic progression in patients with medial compartment osteoarthritis of the knee: results of the two-year multinational knee osteoarthritis structural arthritis study. *Arthritis Rheumat.* **54**, 3494–3507 (2006). 17075851 10.1002/art.22160

[88] Yue, R. et al. Beta-arrestin1 regulates zebrafish hematopoiesis through binding to YY1 and relieving polycomb group repression. *Cell* **139**, 535–546 (2009). 19879840 10.1016/j.cell.2009.08.038

[89] Luo, J. et al. LGR4 is a receptor for RANKL and negatively regulates osteoclast differentiation and bone resorption. *Nat. Med.* **22**, 539–546 (2016). 27064449 10.1038/nm.4076

[90] Pritzker, K. P. et al. Osteoarthritis cartilage histopathology: grading and staging. *Osteoarthr. Cartil.* **14**, 13–29 (2006). 10.1016/j.joca.2005.07.014 16242352

[91] Mori, H. & Cardiff, R. D. Methods of immunohistochemistry and immunofluorescence: converting invisible to visible. *Methods Mol. Biol.* **1458**, 1–12 (2016). 27581010 10.1007/978-1-4939-3801-8_1

[92] Feng, J. et al. Blocking STAT3 by pyrvinium pamoate causes metabolic lethality in KRAS-mutant lung cancer. *Biochem. Pharmacol.* **177**, 113960 (2020). 32298693 10.1016/j.bcp.2020.113960

