# Supplementary material for: PGE2 activates EP4 in subchondral bone osteoclasts to regulate osteoarthritis
Source: Bone Res. 2022 Mar 9;10:27. doi: 10.1038/s41413-022-00201-4 (PMC8904489; doi:10.1038/s41413-022-00201-4)
Supplement: Supplementary file 1 — Supplementary figure [file 41413_2022_201_MOESM1_ESM.pptx]

## Slide 1
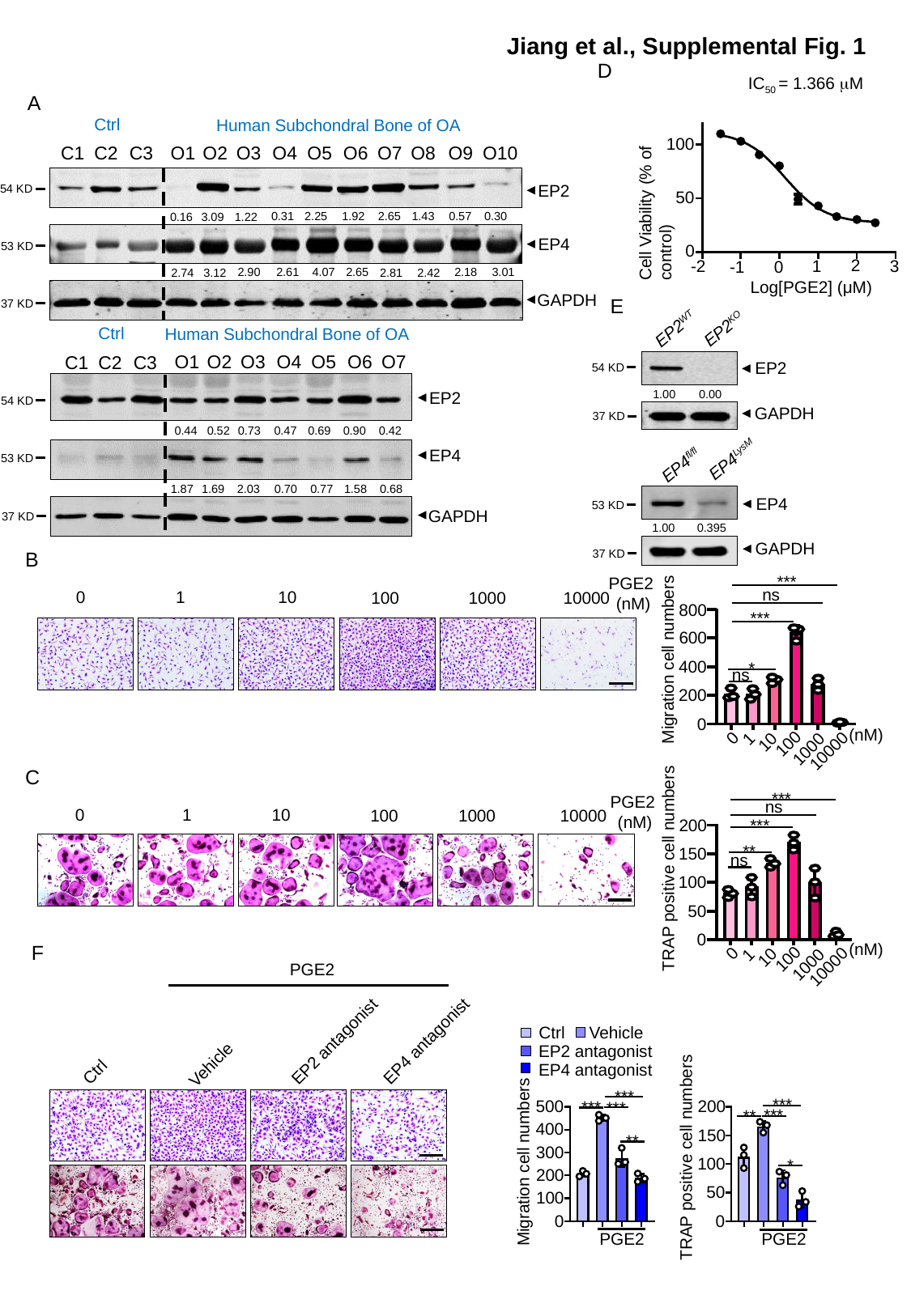

Jiang et al., Supplemental Fig. 1
D
IC50 = 1.366 μM
A
Ctrl
Human Subchondral Bone of OA
100
O10
O1
O2
O3
O7
O8
O9
O4
O5
O6
C1
C2
C3
Cell Viability (% of control)
EP2
 54 KD
50
0.57
0.30
0.31
2.25
1.92
2.65
1.43
3.09
0.16
1.22
EP4
53 KD
0
2
1
3
-2
-1
0
2.18
3.01
2.90
2.61
4.07
2.65
2.81
2.42
3.12
2.74
Log[PGE2] (μM)
GAPDH
E
37 KD
EP2WT
EP2KO
Ctrl
Human Subchondral Bone of OA
O1
O2
O3
O7
O4
O5
O6
C1
C2
C3
EP2
54 KD
0.00
1.00
EP2
54 KD
GAPDH
37 KD
0.73
0.47
0.69
0.90
0.42
0.52
0.44
EP4
EP4LysM
53 KD
EP4fl/fl
2.03
0.70
0.77
1.58
0.68
1.69
1.87
EP4
53 KD
GAPDH
37 KD
1.00
0.395
GAPDH
37 KD
B
***
PGE2
(nM)
ns
10
1
0
10000
1000
100
800
***
600
Migration cell numbers
400
*
ns
200
0
(nM)
0
 1
10
100
1000
10000
C
***
PGE2
(nM)
ns
1
10
0
10000
1000
100
200
***
**
150
ns
TRAP positive cell numbers
100
50
0
(nM)
F
0
 1
10
100
1000
10000
PGE2
Ctrl
Vehicle
EP4 antagonist
EP2 antagonist
EP2 antagonist
Ctrl
Vehicle
EP4 antagonist
***
***
200
500
***
***
***
**
400
150
**
300
TRAP positive cell numbers
Migration cell numbers
100
*
200
50
100
0
0
PGE2
PGE2

## Slide 2
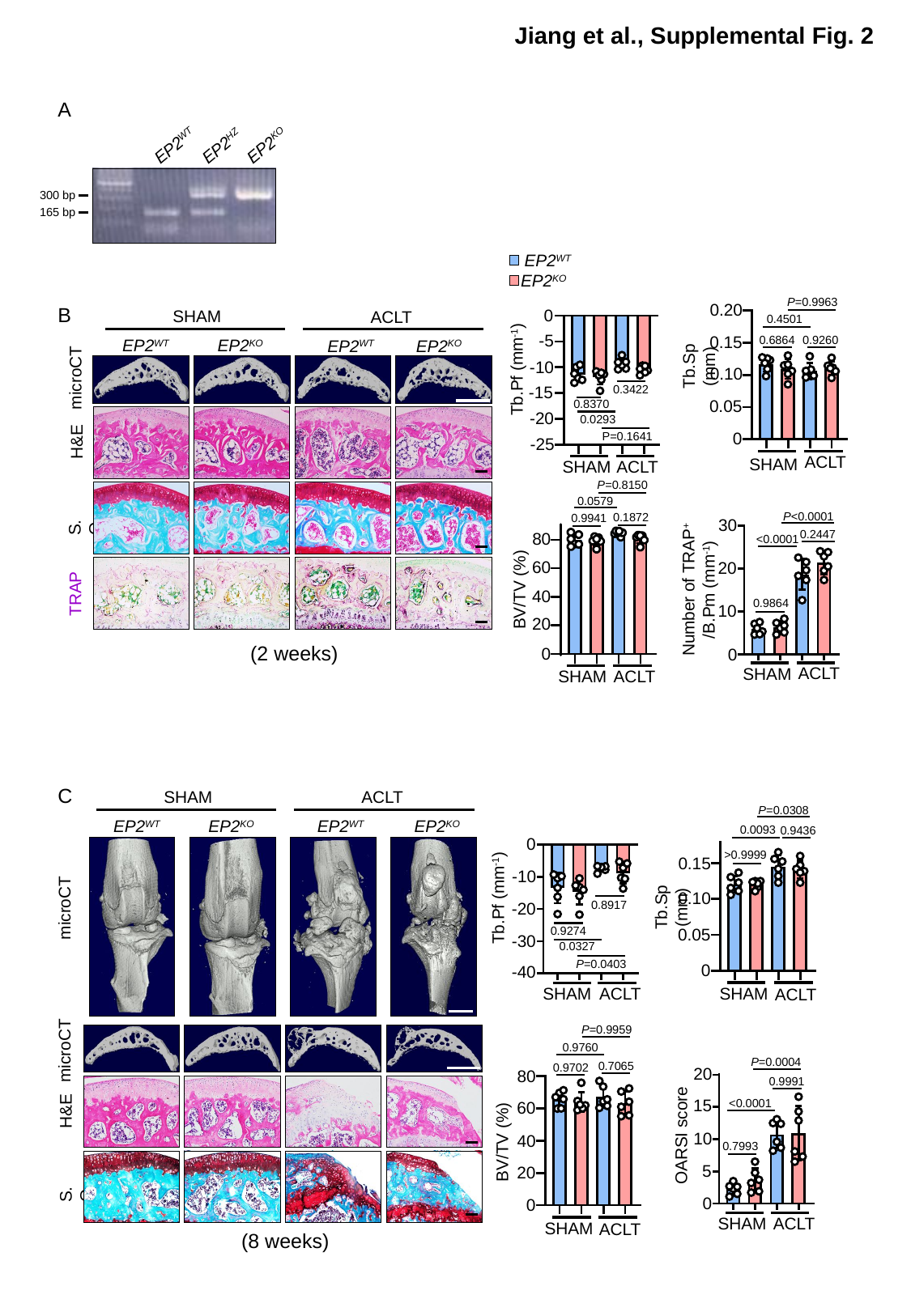

Jiang et al., Supplemental Fig. 2
A
EP2KO
EP2WT
EP2HZ
300 bp
165 bp
EP2WT
 EP2KO
P=0.9963
0.20
B
0
SHAM
ACLT
0.4501
-5
0.15
0.6864
0.9260
 EP2KO
EP2WT
EP2WT
EP2KO
-10
Tb.Sp (mm)
Tb.Pf (mm-1)
0.10
microCT
-15
0.3422
0.05
0.8370
-20
0.0293
0
P=0.1641
H&E
-25
ACLT
SHAM
ACLT
SHAM
P=0.8150
0.0579
S. O.
P<0.0001
0.1872
0.9941
30
0.2447
80
<0.0001
60
20
Number of TRAP+
/B.Pm (mm-1)
BV/TV (%)
TRAP
40
0.9864
10
20
(2 weeks)
0
0
ACLT
SHAM
ACLT
SHAM
C
SHAM
ACLT
P=0.0308
 EP2KO
EP2WT
EP2WT
EP2KO
0.0093
0.9436
0
>0.9999
0.15
-10
0.10
Tb.Pf (mm-1)
microCT
Tb.Sp (mm)
0.8917
-20
0.9274
0.05
-30
0.0327
P=0.0403
0
-40
SHAM
ACLT
SHAM
ACLT
P=0.9959
microCT
0.9760
P=0.0004
0.7065
0.9702
20
80
0.9991
15
<0.0001
60
H&E
OARSI score
10
40
BV/TV (%)
0.7993
5
20
S. O.
0
0
ACLT
SHAM
SHAM
ACLT
(8 weeks)

## Slide 3
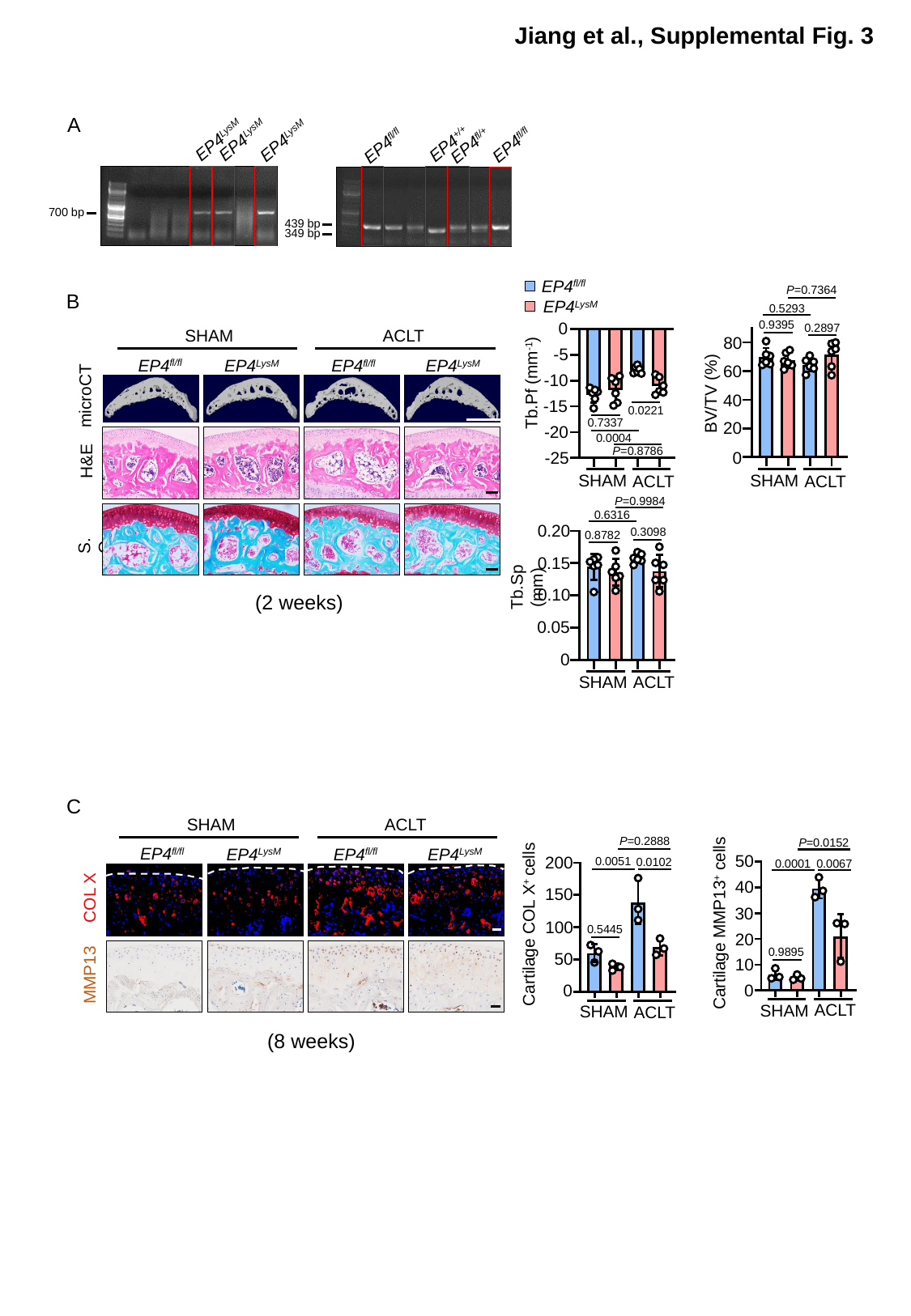

Jiang et al., Supplemental Fig. 3
A
EP4LysM
EP4LysM
EP4LysM
EP4+/+
EP4fl/fl
EP4fl/+
EP4fl/fl
700 bp
439 bp
349 bp
EP4fl/fl
P=0.7364
B
EP4LysM
0.5293
0.9395
0
0.2897
SHAM
ACLT
80
-5
EP4fl/fl
EP4LysM
EP4fl/fl
EP4LysM
60
-10
Tb.Pf (mm-1)
microCT
BV/TV (%)
40
-15
0.0221
0.7337
20
-20
0.0004
P=0.8786
-25
0
H&E
SHAM
SHAM
ACLT
ACLT
P=0.9984
0.6316
0.20
0.3098
S. O.
0.8782
0.15
Tb.Sp (mm)
0.10
(2 weeks)
0.05
0
ACLT
SHAM
C
SHAM
ACLT
P=0.2888
P=0.0152
EP4fl/fl
EP4LysM
EP4fl/fl
EP4LysM
50
200
0.0051
0.0102
0.0067
0.0001
40
COL X
150
30
 Cartilage MMP13+ cells
 Cartilage COL X+ cells
100
0.5445
20
0.9895
50
10
MMP13
0
0
ACLT
SHAM
SHAM
ACLT
(8 weeks)

## Slide 4
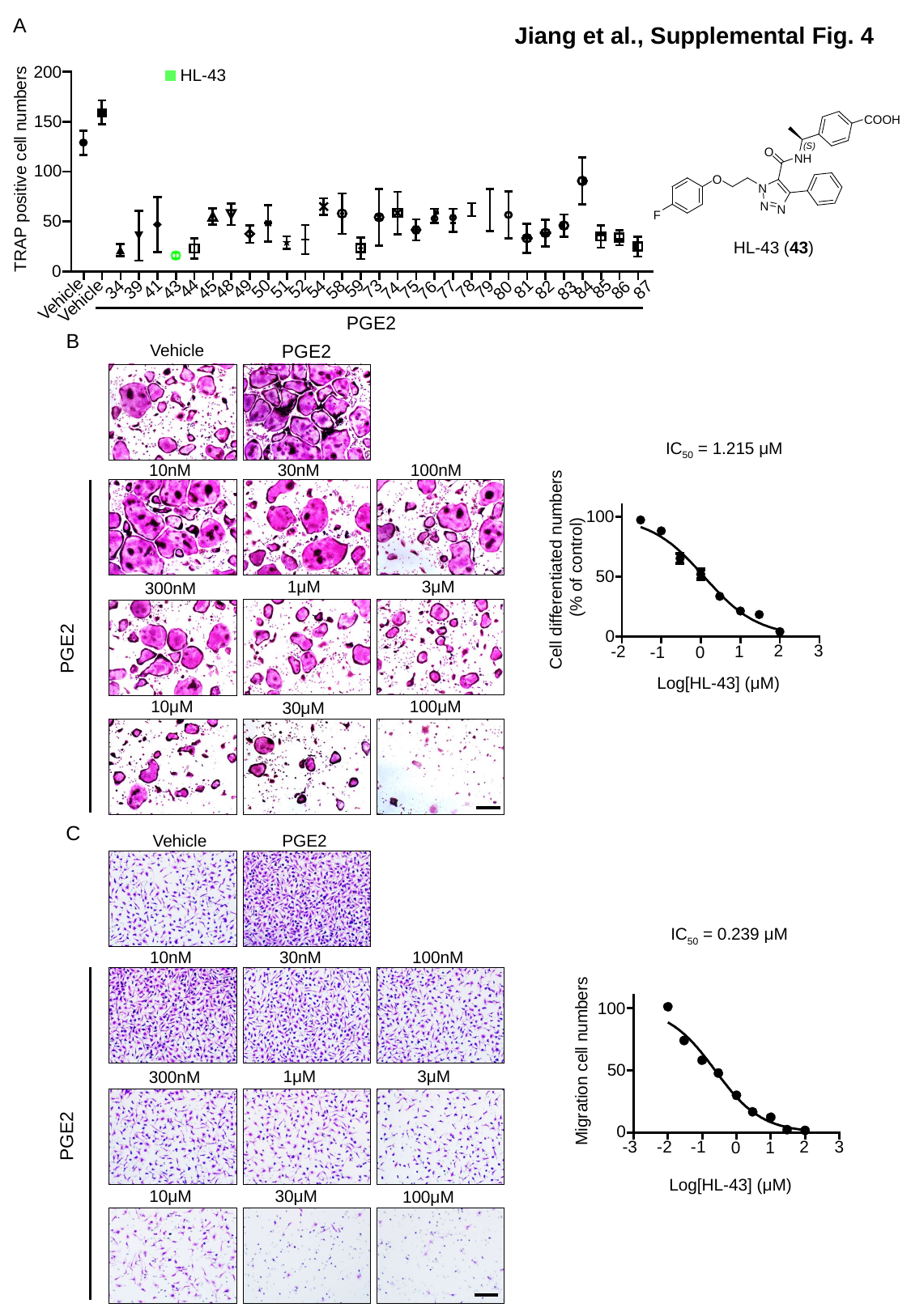

A
Jiang et al., Supplemental Fig. 4
200
HL-43
150
TRAP positive cell numbers
100
50
HL-43 (43)
0
59
44
41
51
48
49
52
81
85
73
78
50
79
54
45
58
39
34
84
87
77
43
75
82
86
80
76
83
74
Vehicle
Vehicle
PGE2
B
Vehicle
PGE2
IC50 = 1.215 μM
100nM
30nM
10nM
100
Cell differentiated numbers
(% of control)
50
1μM
3μM
300nM
0
PGE2
2
3
1
-2
-1
0
Log[HL-43] (μM)
100μM
10μM
30μM
C
Vehicle
PGE2
IC50 = 0.239 μM
100nM
30nM
10nM
100
Migration cell numbers
50
1μM
3μM
300nM
0
PGE2
-3
-2
-1
2
3
0
1
Log[HL-43] (μM)
10μM
30μM
100μM

## Slide 5
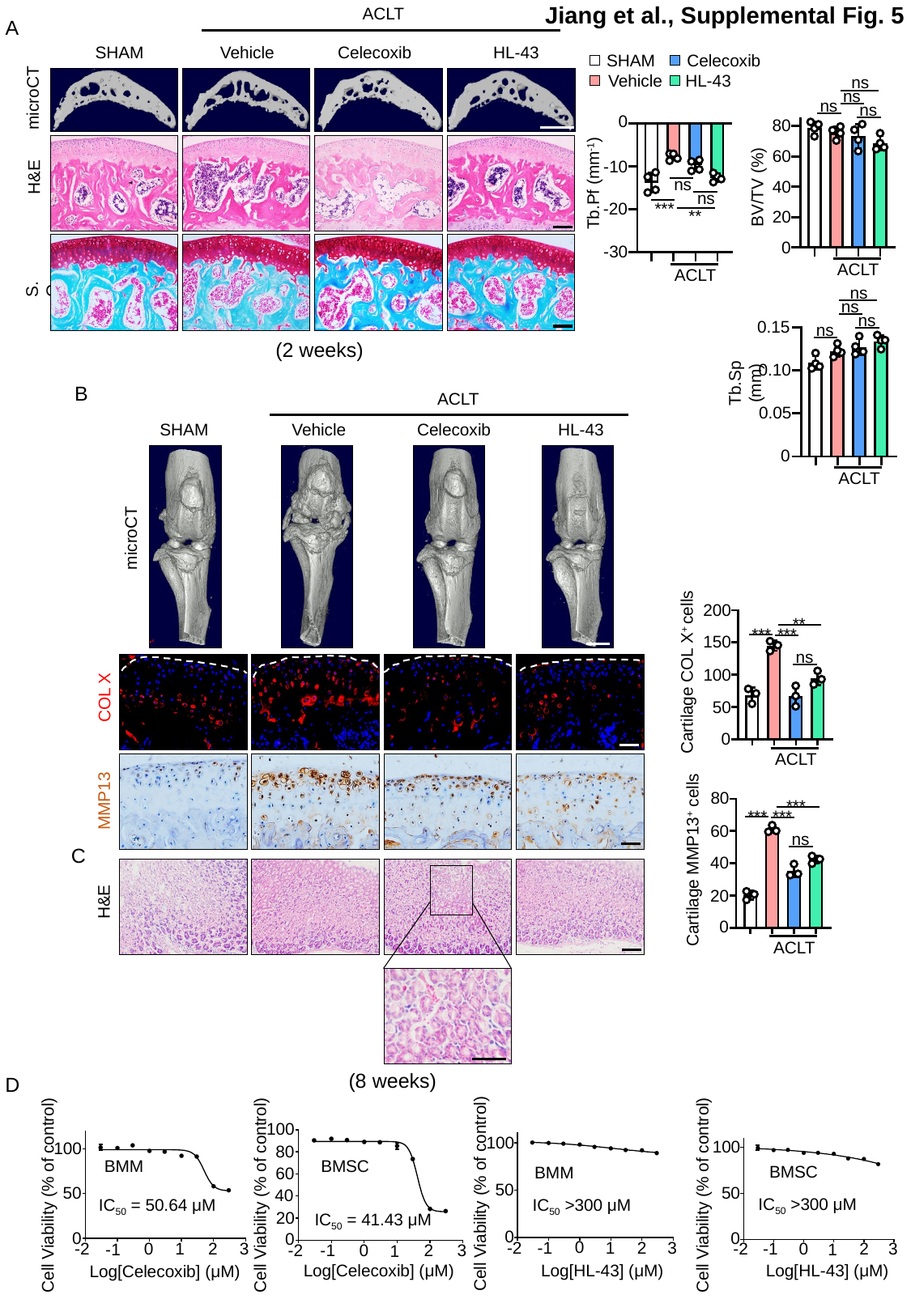

Jiang et al., Supplemental Fig. 5
ACLT
A
Vehicle
SHAM
HL-43
Celecoxib
SHAM
Celecoxib
Vehicle
HL-43
ns
microCT
ns
ns
ns
0
80
60
-10
H&E
Tb.Pf (mm-1)
ns
40
BV/TV (%)
ns
***
-20
**
20
0
-30
ACLT
ACLT
S. O.
ns
ns
ns
0.15
ns
(2 weeks)
0.10
Tb.Sp (mm)
B
ACLT
0.05
HL-43
Vehicle
Celecoxib
SHAM
0
ACLT
microCT
200
**
***
***
150
ns
 Cartilage COL X+ cells
100
COL X
50
0
ACLT
MMP13
80
***
***
***
60
ns
C
 Cartilage MMP13+ cells
40
H&E
20
0
ACLT
(8 weeks)
D
100
100
100
100
80
BMM
BMSC
BMSC
BMM
60
50
50
50
Cell Viability (% of control)
Cell Viability (% of control)
Cell Viability (% of control)
Cell Viability (% of control)
40
IC50 >300 μM
IC50 = 50.64 μM
IC50 >300 μM
20
IC50 = 41.43 μM
0
0
0
0
0
2
3
-2
1
-1
0
1
-2
2
3
0
-1
2
3
-2
-1
1
0
2
3
-1
-2
1
Log[HL-43] (μM)
Log[HL-43] (μM)
Log[Celecoxib] (μM)
Log[Celecoxib] (μM)

## Slide 6
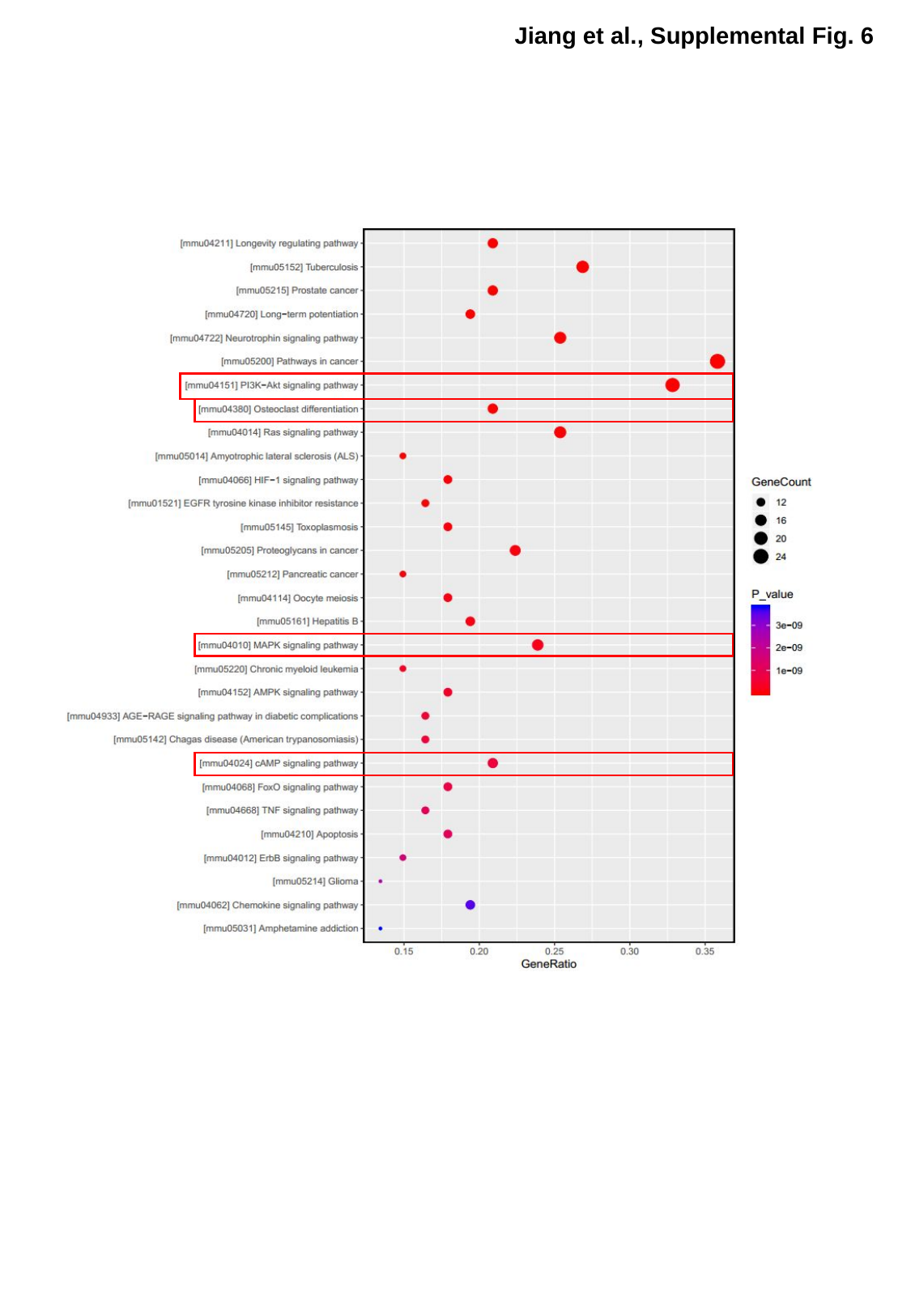

Jiang et al., Supplemental Fig. 6

## Slide 7
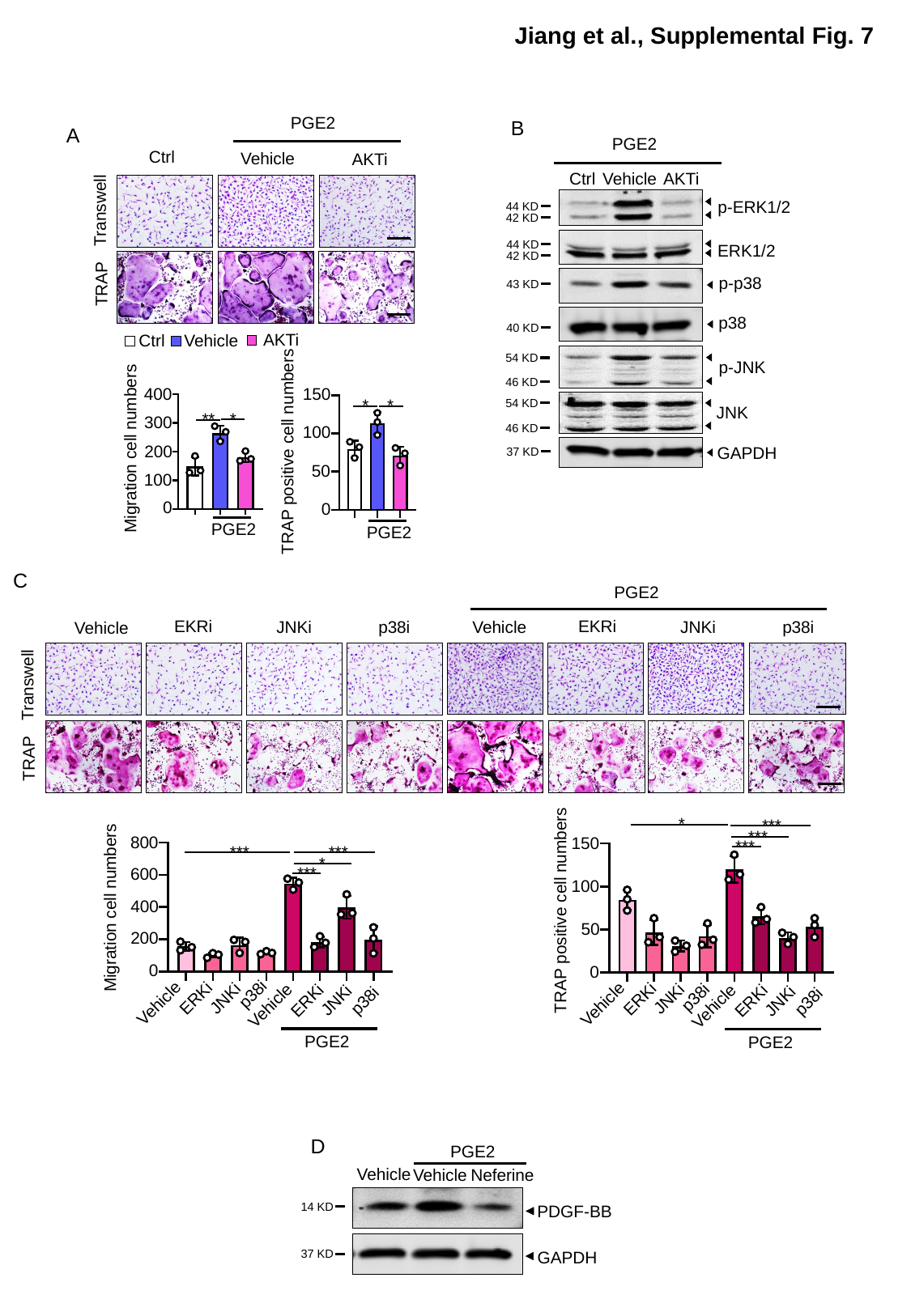

Jiang et al., Supplemental Fig. 7
PGE2
B
A
PGE2
 Ctrl
 Vehicle
AKTi
 Ctrl
 Vehicle
AKTi
 p-ERK1/2
Transwell
44 KD
42 KD
44 KD
 ERK1/2
42 KD
TRAP
 p-p38
43 KD
 p38
40 KD
AKTi
Ctrl
Vehicle
54 KD
p-JNK
46 KD
400
150
*
*
54 KD
 JNK
*
**
300
46 KD
100
Migration cell numbers
TRAP positive cell numbers
200
GAPDH
37 KD
50
100
0
0
PGE2
PGE2
C
PGE2
EKRi
EKRi
Vehicle
p38i
p38i
JNKi
JNKi
Vehicle
Transwell
TRAP
*
***
***
800
150
***
***
***
*
***
600
100
400
Migration cell numbers
TRAP positive cell numbers
50
200
0
0
p38i
p38i
ERKi
JNKi
ERKi
JNKi
ERKi
JNKi
p38i
ERKi
JNKi
p38i
Vehicle
Vehicle
Vehicle
Vehicle
PGE2
PGE2
D
PGE2
 Vehicle
 Vehicle
Neferine
14 KD
PDGF-BB
37 KD
GAPDH

## Slide 8
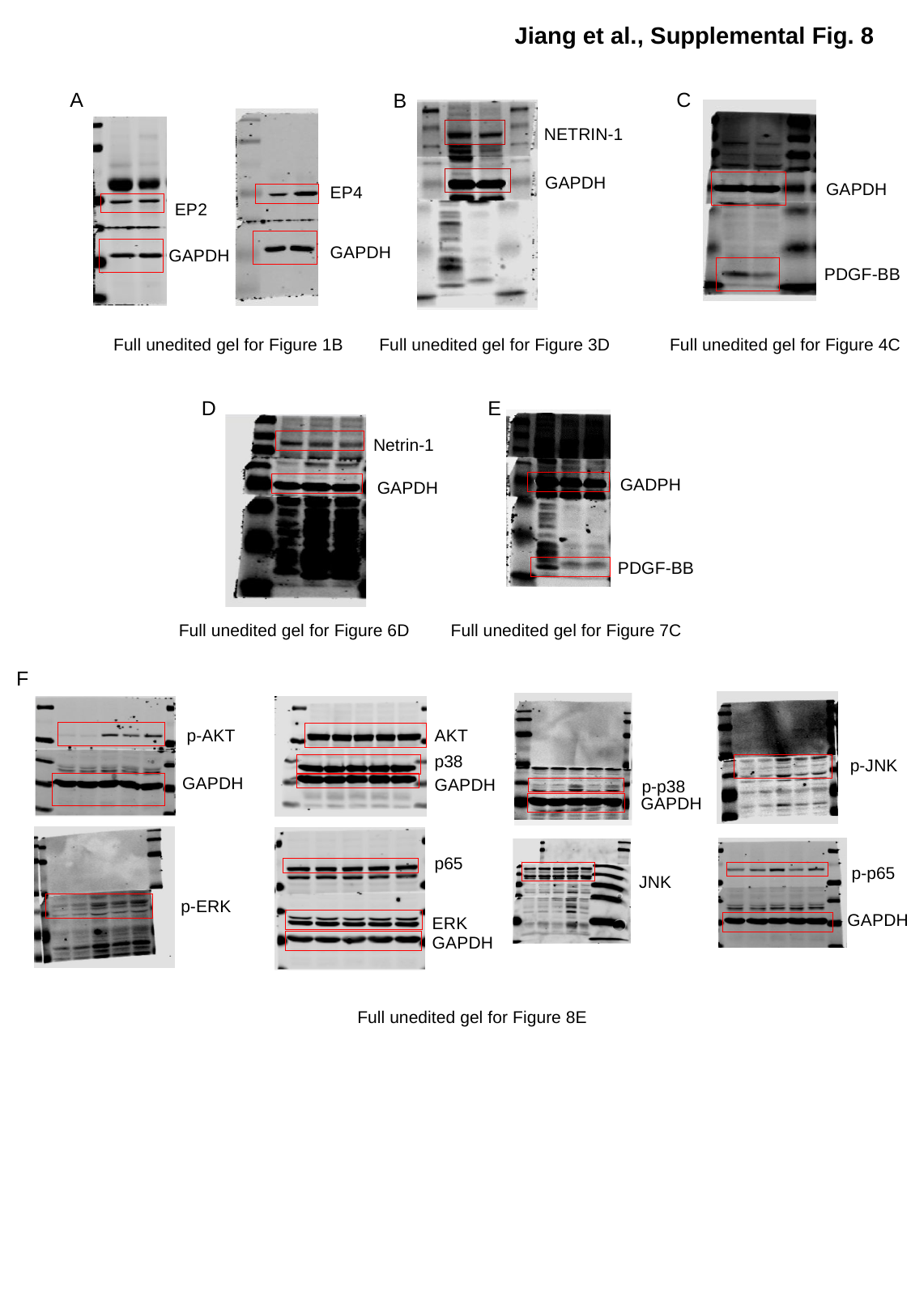

Jiang et al., Supplemental Fig. 8
C
A
B
NETRIN-1
GAPDH
GAPDH
EP4
EP2
GAPDH
GAPDH
PDGF-BB
Full unedited gel for Figure 1B
Full unedited gel for Figure 3D
Full unedited gel for Figure 4C
D
E
Netrin-1
GADPH
GAPDH
PDGF-BB
Full unedited gel for Figure 6D
Full unedited gel for Figure 7C
F
p-AKT
AKT
p38
p-JNK
GAPDH
GAPDH
p-p38
GAPDH
p65
p-p65
JNK
p-ERK
GAPDH
ERK
GAPDH
Full unedited gel for Figure 8E

## Slide 9
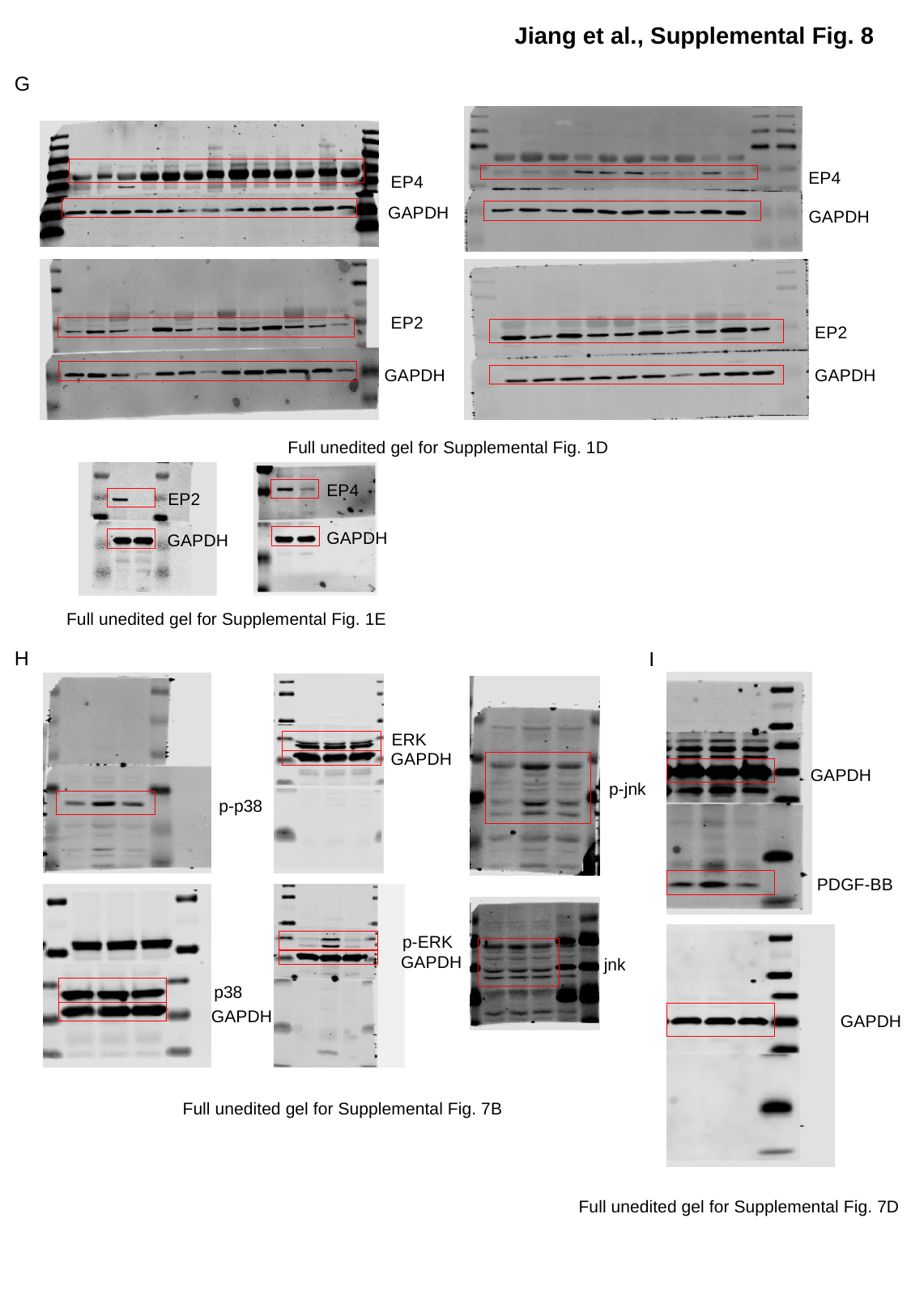

Jiang et al., Supplemental Fig. 8
G
EP4
EP4
GAPDH
GAPDH
EP2
EP2
GAPDH
GAPDH
Full unedited gel for Supplemental Fig. 1D
EP4
EP2
GAPDH
GAPDH
Full unedited gel for Supplemental Fig. 1E
H
I
ERK
GAPDH
GAPDH
p-jnk
p-p38
PDGF-BB
p-ERK
GAPDH
jnk
p38
GAPDH
GAPDH
Full unedited gel for Supplemental Fig. 7B
Full unedited gel for Supplemental Fig. 7D
