# Supplementary material for: PGE2 activates EP4 in subchondral bone osteoclasts to regulate osteoarthritis
Source: Bone Res. 2022 Mar 9;10:27. doi: 10.1038/s41413-022-00201-4 (PMC8904489; doi:10.1038/s41413-022-00201-4)
Supplement: Supplementary file 2 — Supplemental Figure [file 41413_2022_201_MOESM2_ESM.pdf]

Supplemental Figure Legend

Supplemental Figure 1. PGE2 regulates migration and osteoclast differentiation

(A) The protein expression levels of EP2 and EP4 in control (Ctrl, n = 3) and human subchondral bone of OA patients (n = 17). The experiment was performed in three biological replicates.

(B) PGE2 regulated osteoclast migration. Representative images of the migrated BMMs, stimulated with 10 ng/ml M-CSF, 50 ng/ml RANKL and different concentrations of PGE2, from WT mice were photographed (left) and quantified (right). Error bars are mean ± s.d. *P < 0.05, and ***P < 0.01, ns, not significant by one-way ANOVA followed by Tukey's t-tests. The experiment was performed in three biological replicates.

(C) PGE2 regulated osteoclast differentiation. Representative images of the differentiated osteoclast cells were photographed (left) and quantified (right). Error bars are mean ± s.d. *P < 0.05, and ***P < 0.01, ns, not significant by one-way ANOVA followed by Tukey's t-tests. The experiment was performed in three biological replicates.

(D) The cytotoxicity of PGE2 on primary cultured BMM cells. Primary cultured BMMs were treated with 10 ng/ml M-CSF and indicated concentrations of PGE2 for 48 hours. IC_{50} values were calculated by GraphPad Prism 8.0 software. The experiment was performed in three biological replicates.

(E) Confirmation of gene knockout for *EP2* and *EP4* in BMMs. Western blotting was

used to validate the protein expression of EP2 and EP4 in $EP2^{KO}$ and $EP4^{LysM}$ respectively, compared to the corresponding littermate controls of $EP2^{WT}$ and $EP4^{fl/fl}$.

(F) EP2 and EP4 antagonists inhibit PGE2 induced migration and osteoclast differentiation. BMMs from WT were used to generate osteoclasts by stimulating with 10 ng/ml M-CSF and 50 ng/ml RANKL, and treated EP2 antagonist PF-04418948 (2 μM) or EP4 antagonist Grapiprant (10 μM). Representative image of cells from transwell migration assay (Transwell), and osteoclasts differentiation assay (TRAP staining) (left), and the corresponding quantitative analysis (right). Error bars are mean ± s.d. *$P < 0.05$, **$P < 0.01$ and ***$P < 0.01$ by one-way ANOVA followed by Tukey's t-tests. Scale bars, 50 μm. The experiment was performed in three biological replicates.

Supplemental Figure 2. *EP2* deletion had little effect on OA progression in a murine model of OA

(A) Genotyping validation of EP2 knockout in $EP2^{KO}$ mice compared to $EP2^{WT}$ and littermate. The $EP2^{KO}$ band size is 300bp and the $EP2^{WT}$ band size is 165bp.

(B and C) Representative 3D reconstructed microCT images, hematoxylin and eosin (H&E) and Safranin O-Fast green (S.O.) staining of sagittal sections of articular cartilage of $EP2^{WT}$ and $EP2^{KO}$ mice 2 weeks (B) and 8 weeks (C) post ACLT surgery. Quantitative analysis of structural parameters of subchondral bone including Trabecular pattern factor (Tb.Pf), Trabecular separation (Tb.Sp), Percent bone volume (BV/TV) are presented alongside. Error bars are mean ± s.d. Two-way ANOVA followed by Tukey's t-tests. $n = 6$ for each group. Scale bars, 1 mm (microCT), 50 μm (H&E), 50

μm (S.O.) and. 50 μm (TRAP).

Supplemental Figure 3. *EP4* deletion in osteoclasts inhibits OA progression in a murine model of OA (refer to Fig. 2).

(A) Genotyping validation of EP4 knockout in $EP4^{LysM}$ mice $EP4^{fl/fl}$ littermate. The cre band size is 700bp (left) and the flox band size is 439bp (right).

(B) Representative 3D reconstructed microCT images, hematoxylin and eosin (H&E) and Safranin O-Fast green (S.O.) staining of sagittal sections of articular cartilage of $EP4^{fl/fl}$ or littermate $EP4^{LysM}$ mice 2 weeks after ACLT surgery (left). Quantitative analysis of structural parameters of subchondral bone including Trabecular pattern factor (Tb.Pf), Trabecular separation (Tb.Sp), Percent bone volume (BV/TV) are presented alongside. Error bars are mean ± s.d. Two-way ANOVA followed by Tukey's t-tests. *n* = 6 for each group. Scale bars, 1 mm (microCT), 50 μm (H&E) and 50 μm (S.O.).

(C) Representative images for IHF staining of ColX (red), and IHC staining of Mmp13 in articular cartilage from $EP4^{fl/fl}$ or littermate $EP4^{LysM}$ mice 8 weeks post ACLT surgery (left), and quantitative analysis (right). Scale bars, 20 μm. Error bars are mean ± s.d. Two-way ANOVA followed by Tukey's t-tests. *n* = 3 for each group. Scale bars, 20 μm.

Supplemental Figure 4. Identification of a novel potent EP4 antagonist HL-43 for PGE2-induced migration and osteoclast differentiation.

(A) Screening of EP4 antagonists on PGE2-induced osteoclast differentiation. BMMs

from WT mice were used to generate osteoclasts by stimulating with 10 ng/ml M-CSF and 50 ng/ml RANKL, 100 nM PGE2 and different EP4 specific antagonists for 5 days (left). The experiments were performed in three technical replicates. The chemical structure of HL-43 (right).

(B and C) The inhibitory effects of HL-43 on PGE2-induced osteoclast differentiation (B) and migration (C). Error bars are mean ± s.d. The experiment was performed in three biological replicates. IC_{50} values were calculated by GraphPad Prism 8.0 software.

Supplemental Figure 5. EP4 antagonist HL-43 inhibits OA progression in a murine model of OA with low gastrointestinal toxicity (refer to Fig. 5).

(A) Representative 3D reconstructed microCT images, hematoxylin and eosin (H&E) and Safranin O-Fast green (S.O.) staining of sagittal sections of articular cartilage from WT mice treated with celecoxib (30 mg/kg) or HL-43 (30 mg/kg) 2 weeks post ACLT surgery (left). Quantitative analysis of structural parameters of subchondral bone including Trabecular pattern factor (Tb.Pf), Trabecular separation (Tb.Sp), Percent bone volume (BV/TV) are presented alongside. Error bars are mean ± s.d. *$P < 0.05$, ns, not significant by one-way ANOVA followed by Tukey's t-tests. $n = 4$ for each group. Scale bars, 1 mm (microCT), 50 μm (H&E) and 50 μm (S.O.).

(B) Representative 3D reconstructed microCT images of knee joint, IHF for ColX, and IHC for Mmp13 from WT mice treated with indicated celecoxib (30 mg/kg) or HL-43 (30 mg/kg) 8 weeks post ACLT surgery. microCT, $n = 17$ for SHAM group, $n = 21$ for vehicle treated group, $n = 18$ for celecoxib treated group, $n = 18$ for HL-43 treated

group. Quantitative analysis for IHF of ColX and IHC of Mmp13 (right), $n = 3$ for each group. Error bars are mean ± s.d. $^{**}P < 0.01$ and $^{***}P < 0.001$, ns, not significant by one-way ANOVA followed by Tukey's t-tests. Scale bars, 1 mm (microCT), 20 μm (IHF and IHC).

(C) Representative H&E staining of stomach sections from WT mice treated with celecoxib (30 mg/kg) or HL-43 (30 mg/kg) 8 weeks post ACLT surgery. Scale bars, 50 μm.

(D) The toxicity of celecoxib and HL-43 in BMMs and BMSCs. Primary BMMs or BMSCs were treated with indicated concentrations of celecoxib or HL-43 for 48 hour and cell viability was examined using the cell proliferation assay kit. IC_{50} values were calculated by GraphPad Prism 8.0 software. The experiment was performed in three biological replicates.

Supplemental Figure 6. Differential phosphoprotein expression between *EP4^{LysM}* and *EP4$^{fl/fl}$* osteoclast cells using the phosphoproteome antibody array.

BMMs from *EP4^{LysM}* and *EP4$^{fl/fl}$* mice were used to generate osteoclasts by using 10 ng/ml M-CSF and 50 ng/ml RANKL, and incubated with 100 nM PGE2 for 3 hours. Subsequently, the cell lysates were used to detect phosphorylated proteins with an antibody array. The levels of the individual proteins were normalized to total protein levels, and differentially regulated proteins were subjected to KEGG pathway enrichment analysis.

Supplemental Figure 7. PGE2 regulates migration and osteoclast differentiation through ATK/MAPK signalling, and PDGF-BB expression via NF-κB signalling.

(A) AKT inhibitor GSK2141795 inhibited PGE2 induced migration and osteoclast differentiation. BMMs from WT were used to generate osteoclasts by stimulating with 10 ng/ml M-CSF, 50 ng/ml RANKL and 100nM PGE2, with or without GSK2141795 (10 μM). Representative image of cells from transwell migration assay (Transwell), and osteoclasts differentiation assay (TRAP staining) (top), and the corresponding quantitative analysis (bottom). Error bars are mean ± s.d. $^{*}P < 0.05$ and $^{**}P < 0.01$ by one-way ANOVA followed by Tukey's t-tests. Scale bars, 50 μm. The experiment was performed in three biological replicates.

(B) Representative images of indicated protein expression by western blotting for osteoclasts generated using BMMs from WT mice, and treated with or without PGE2 and AKT inhibitor GSK2141795 for 3 hours. The experiment was performed in three biological replicates.

(C) Representative images of migration and osteoclast differentiation in using BMMs from WT mice stimulated with 10 ng/ml M-CSF and 50 ng/ml RANKL. The cells were treated with different MAPKs inhibitors (AZD6244: 10 μM, JNK-IN-8: 1 μM and SB203580: 2 μM), with or without PGE2 stimulation. Graphs from the image analyses are presented underneath. Error bars are mean ± s.d. $^{*}P < 0.05$, $^{**}P < 0.01$ and $^{***}P < 0.001$ by one-way ANOVA followed by Tukey's t-tests. Scale bars, 50 μm. The experiment was performed in three biological replicates.

(D) Representative image of western blotting of PDGF-BB in primary osteoclasts

generated from WT BMMs. The BMMs were stimulated with either 10 ng/ml M-CSF, 50 ng/ml RANKL osteoclastogenic media alone, or with PGE2 (100 nM), or ith PGE 2 and Neferine (5 μM; NF-κB inhibitor) for 3 days. The experiment was performed in three biological replicates.

Supplemental Figure 8. Whole gel images for western blots.

Jiang et al., Supplemental Fig. 1

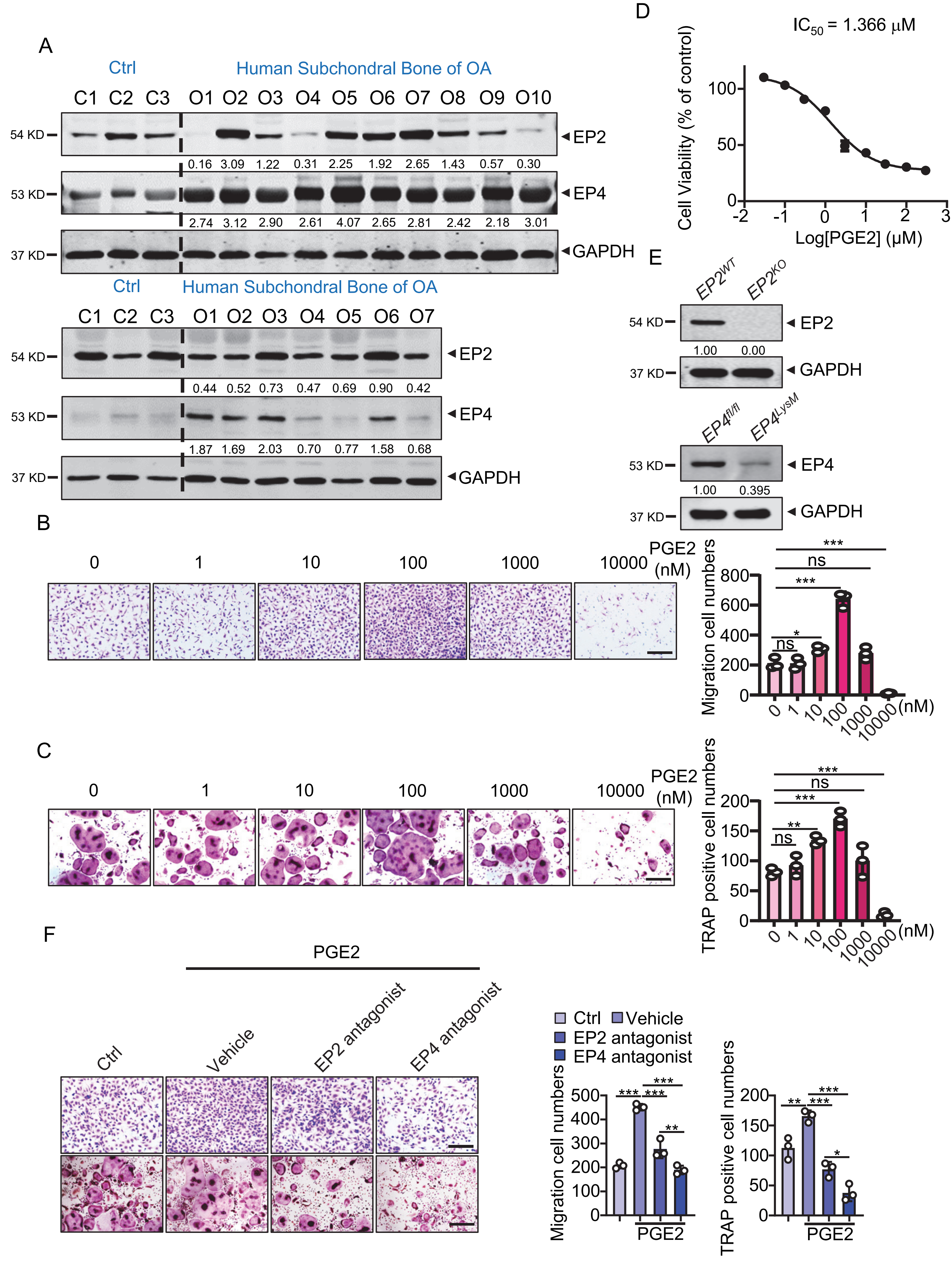

Jiang et al., Supplemental Fig. 2

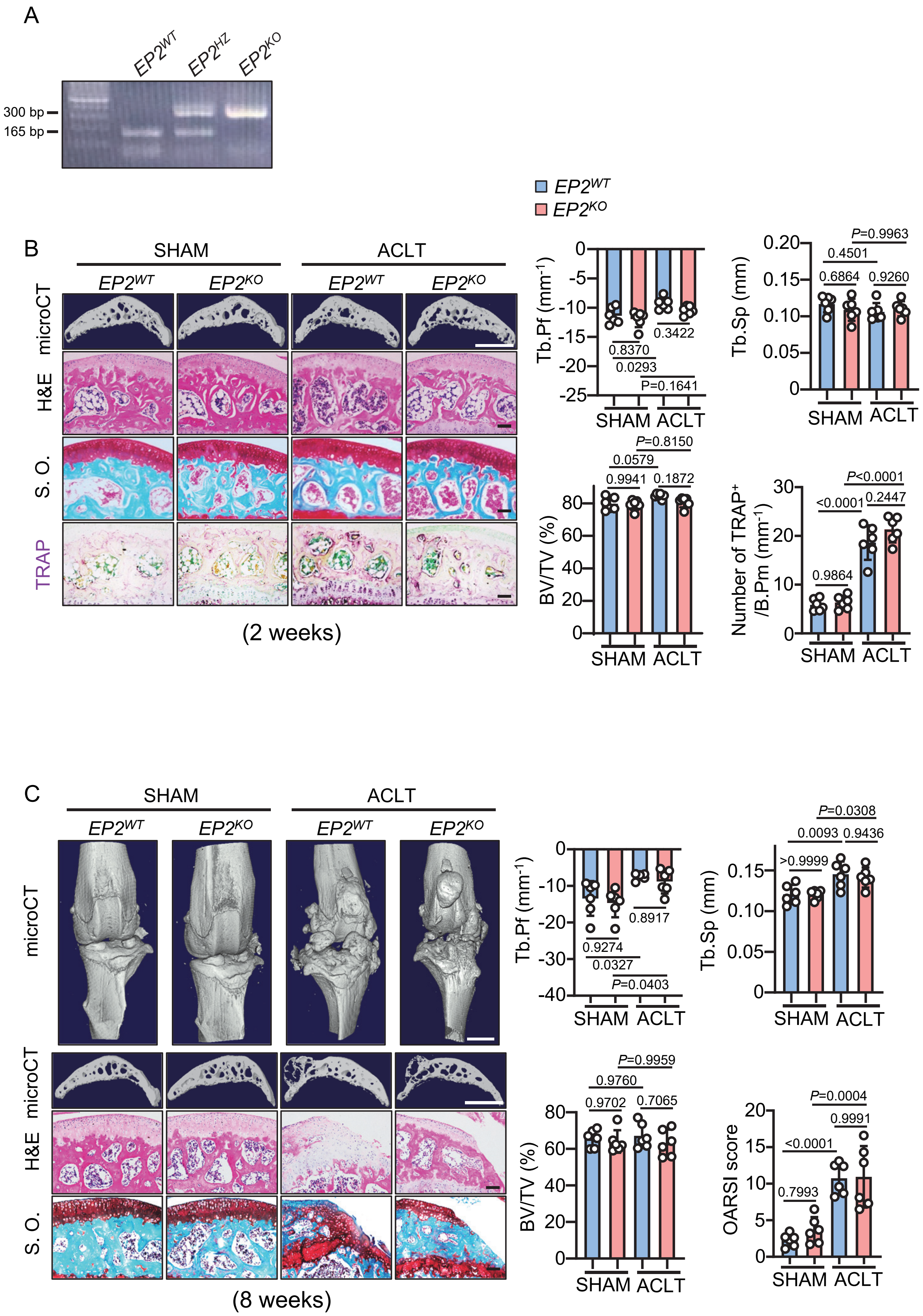

Jiang et al., Supplemental Fig. 3

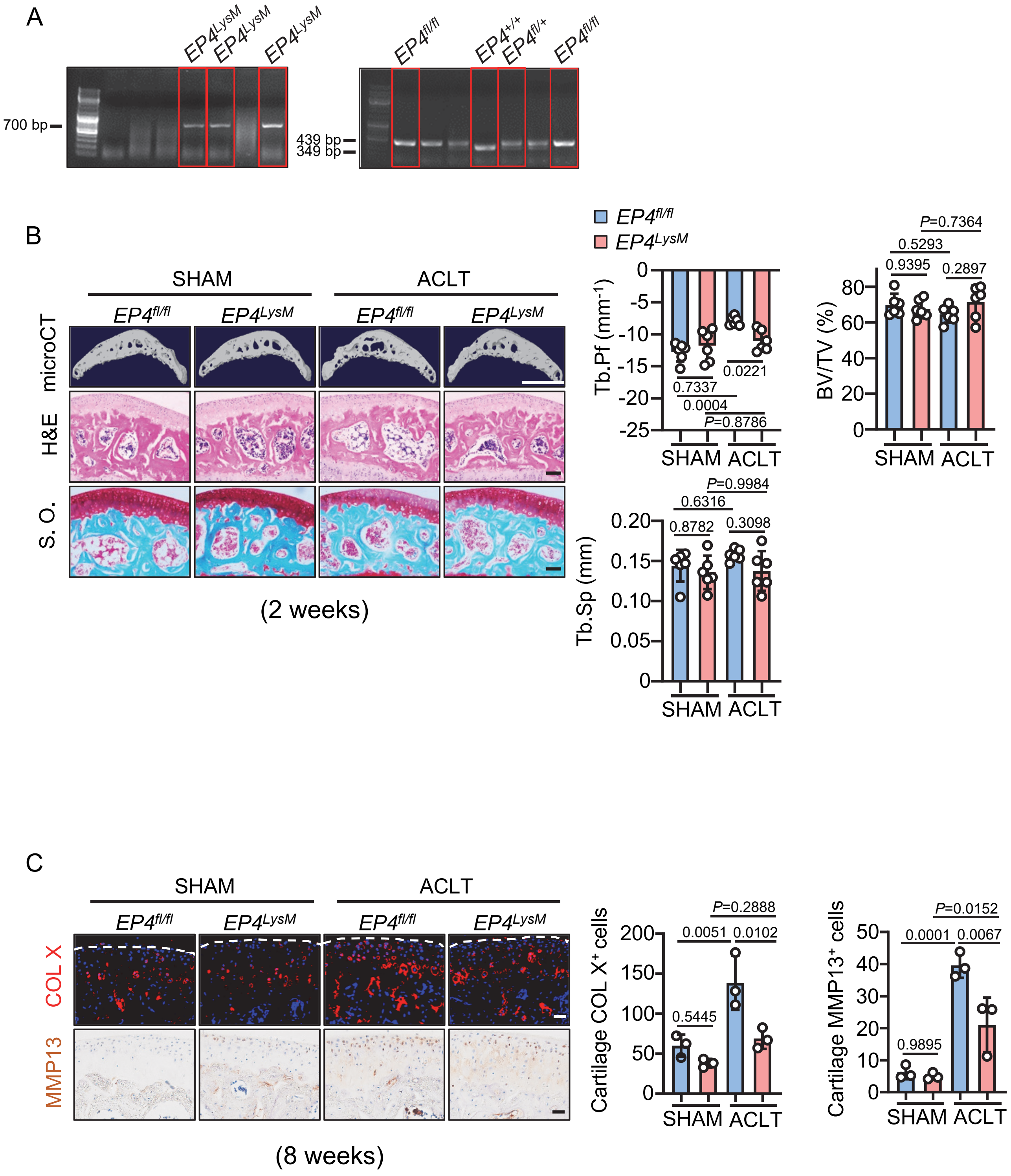

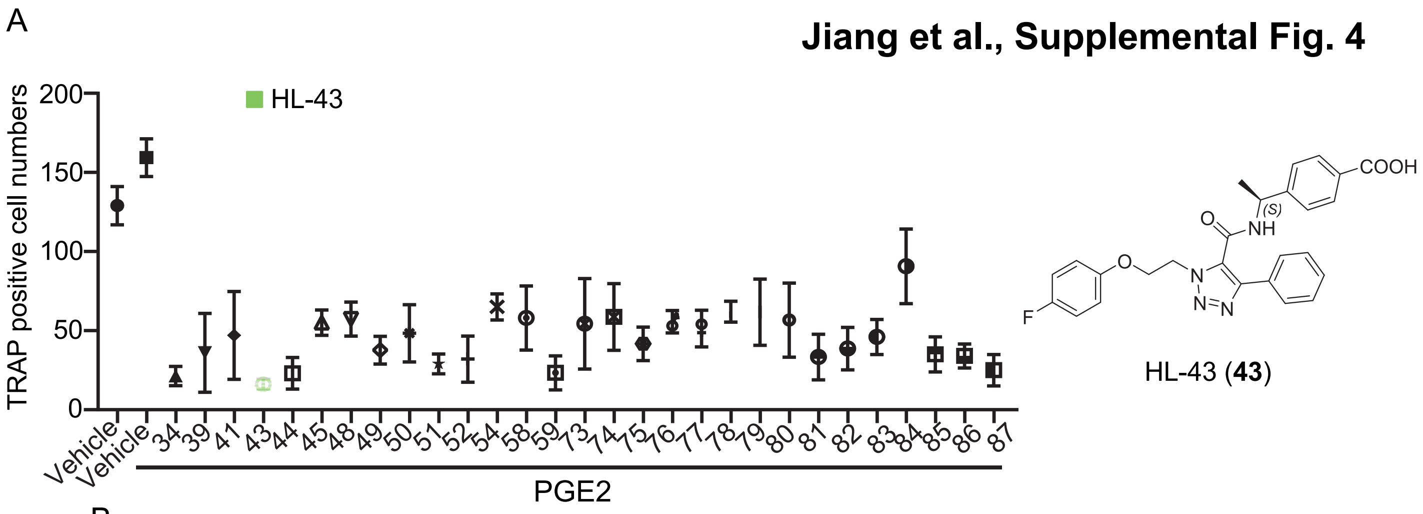
A
HL-43
TRAP positive cell numbers
200
150
100
50
0
Vehicle
Vehicle
34 39 41 43 44 45 48 49 50 51 52 54 58 59 73 74 75 76 77 78 79 80 81 82 83 84 85 86 87
PGE2
COOH
O
NH
(S)
O
N
N=N
F
HL-43 (43)

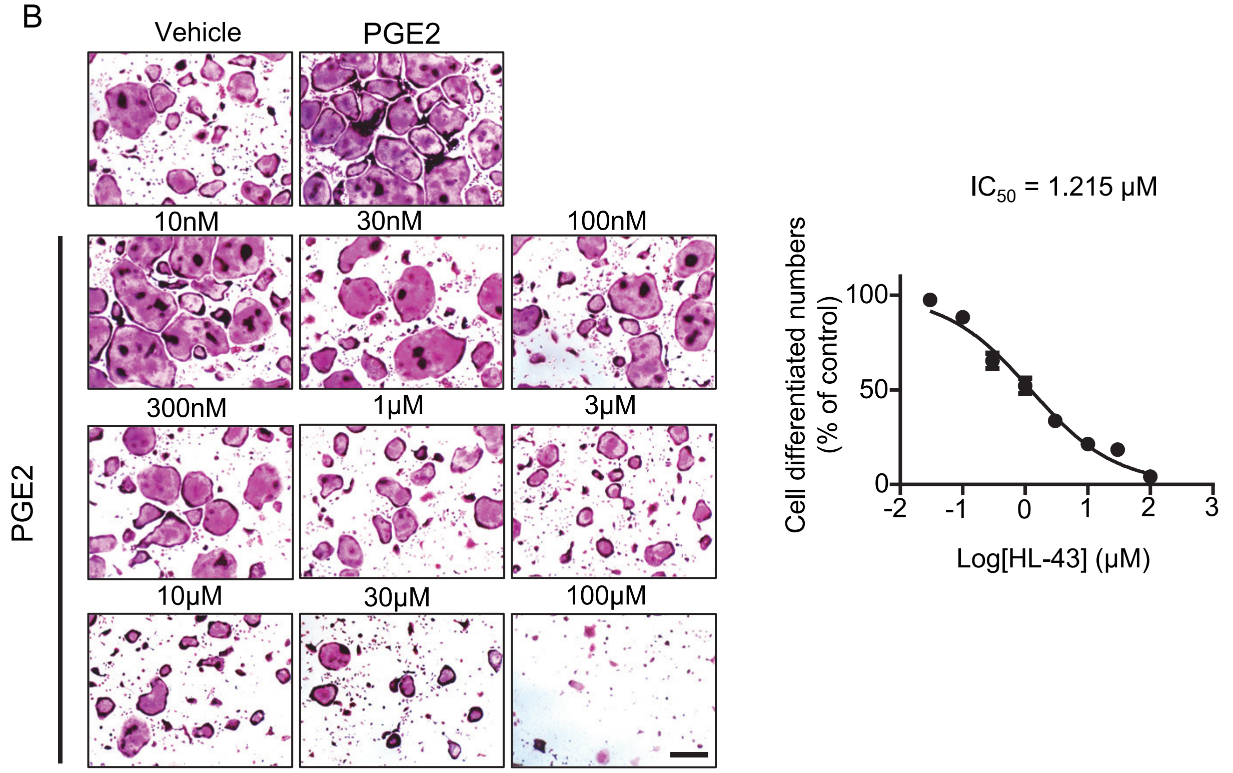
B
Vehicle
PGE2
10nM
30nM
100nM
300nM
1μM
3μM
10μM
30μM
100μM
PGE2
IC50 = 1.215 μM
Cell differentiated numbers (% of control)
Log[HL-43] (μM)

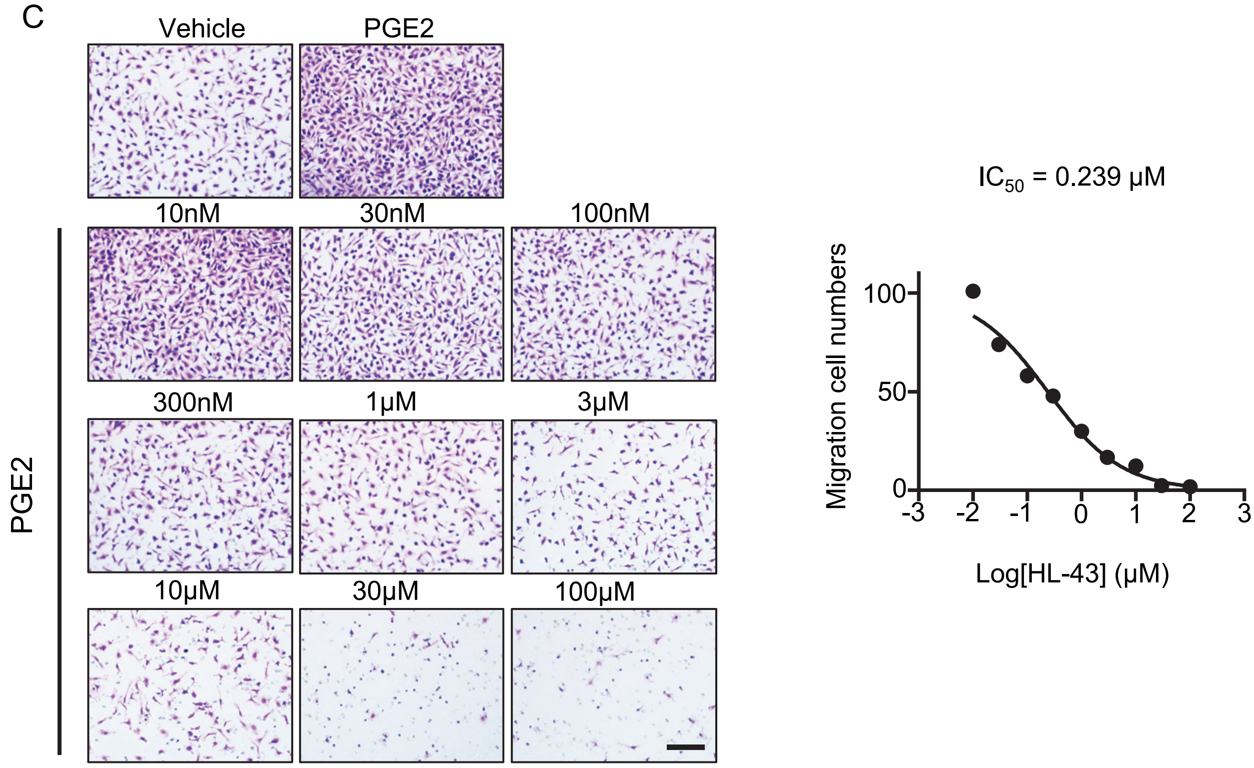
C
Vehicle
PGE2
10nM
30nM
100nM
300nM
1μM
3μM
10μM
30μM
100μM
PGE2
IC50 = 0.239 μM
Migration cell numbers
Log[HL-43] (μM)

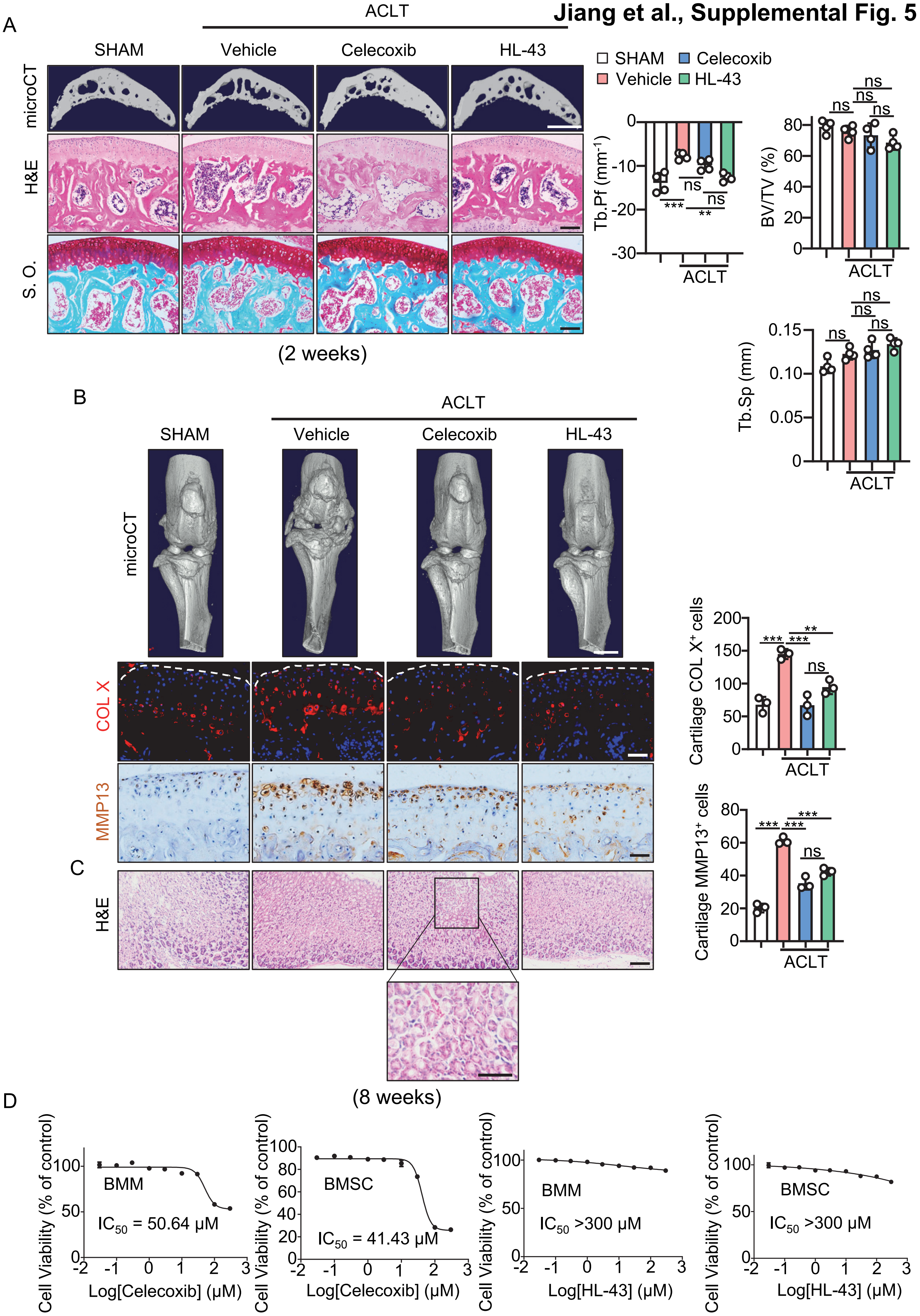
A
ACLT
SHAM
Vehicle
Celecoxib
HL-43
microCT
H&E
S. O.
(2 weeks)
SHAM
Vehicle
Celecoxib
HL-43
Tb.Pf (mm-1)
ns
ns
***
**
ACLT
BV/TV (%)
ns
ns
ns
ns
ACLT
Tb.Sp (mm)
ns
ns
ns
ns
ACLT
B
ACLT
SHAM
Vehicle
Celecoxib
HL-43
microCT
COL X
MMP13
C
H&E
(8 weeks)
Cartilage COL X+ cells
***
***
**
ns
ACLT
Cartilage MMP13+ cells
***
***
***
ns
ACLT
D
Cell Viability (% of control)
BMM
IC50 = 50.64 μM
Log[Celecoxib] (μM)
BMSC
IC50 = 41.43 μM
Log[Celecoxib] (μM)
BMM
IC50 >300 μM
Log[HL-43] (μM)
BMSC
IC50 >300 μM
Log[HL-43] (μM)

Jiang et al., Supplemental Fig. 6

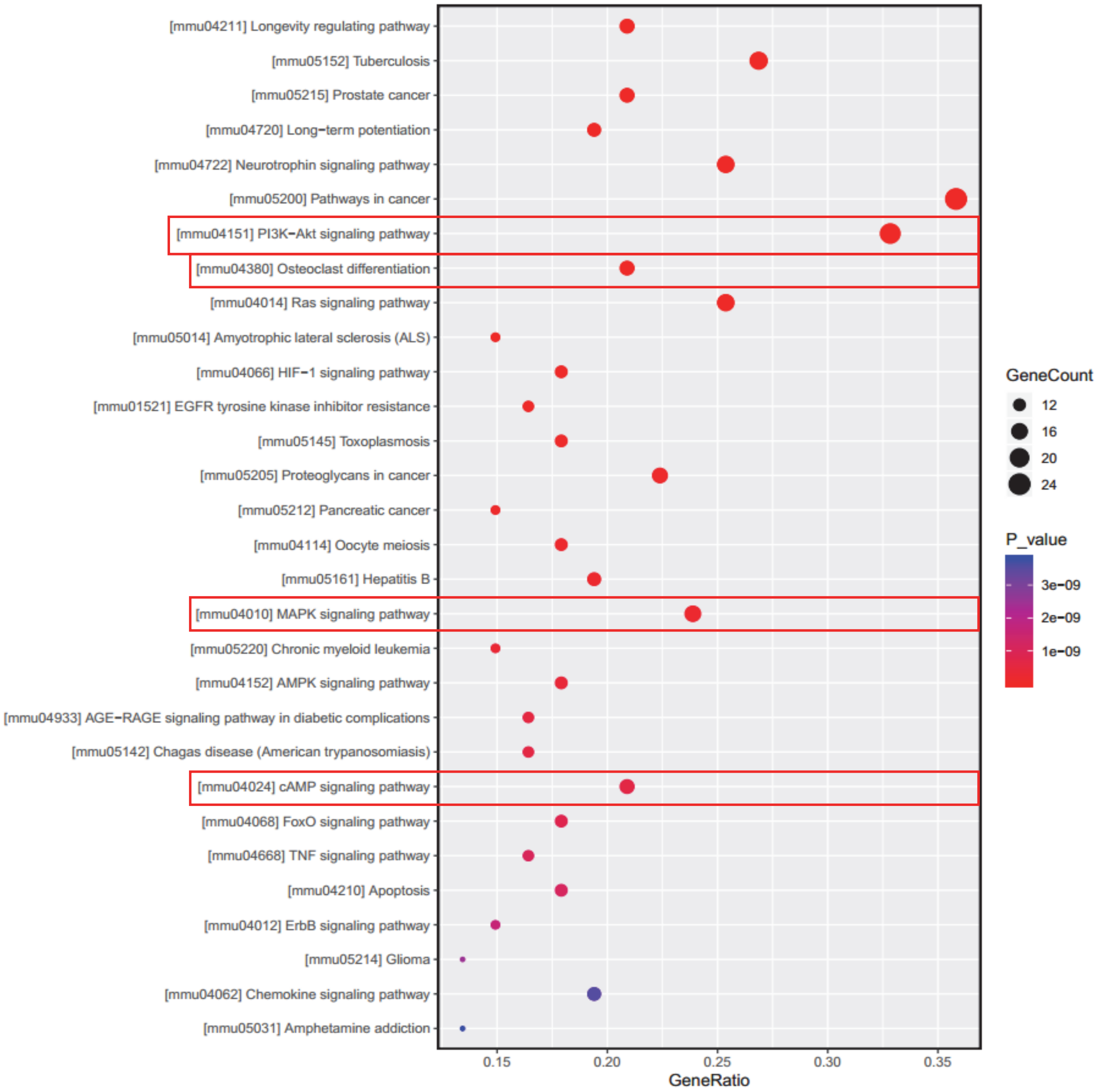

Jiang et al., Supplemental Fig. 7

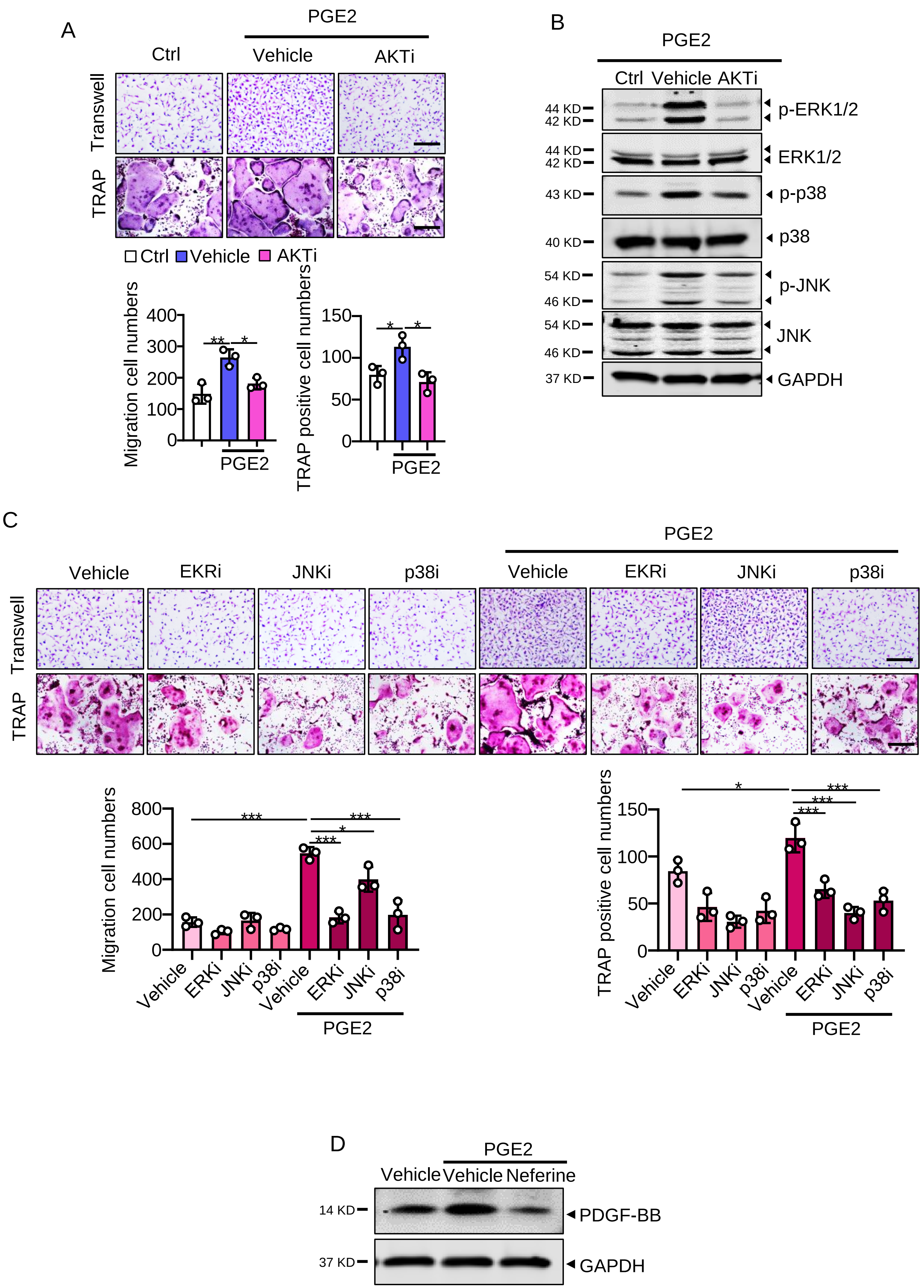

Jiang et al., Supplemental Fig. 8

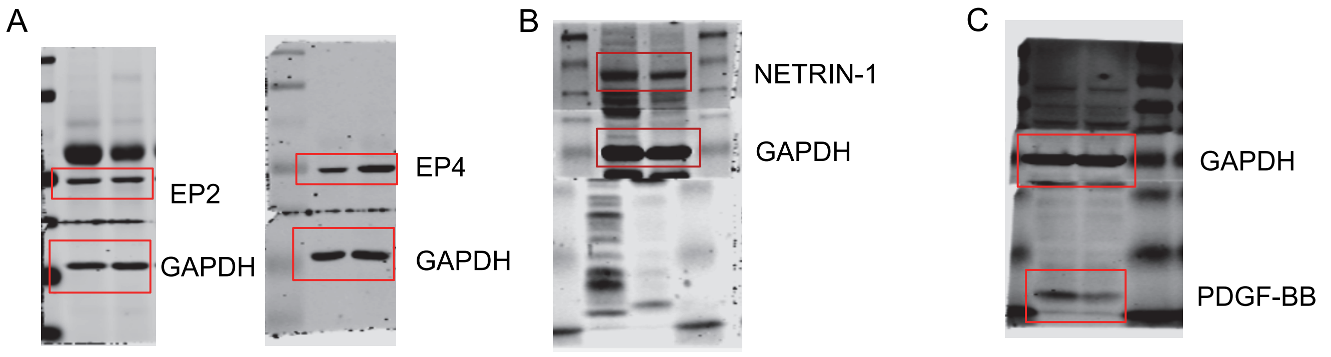

Full unedited gel for Figure 1B Full unedited gel for Figure 3D Full unedited gel for Figure 4C

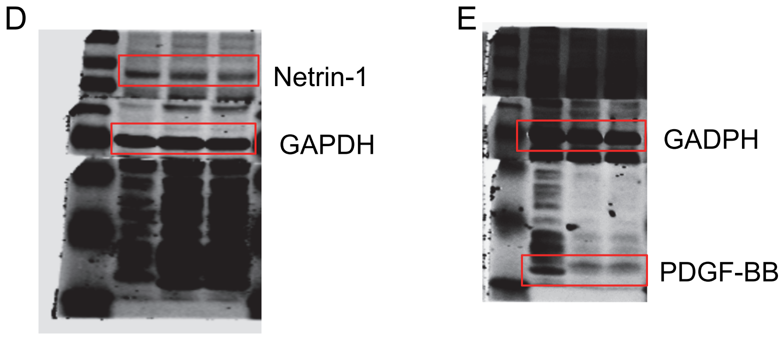

Full unedited gel for Figure 6D Full unedited gel for Figure 7C

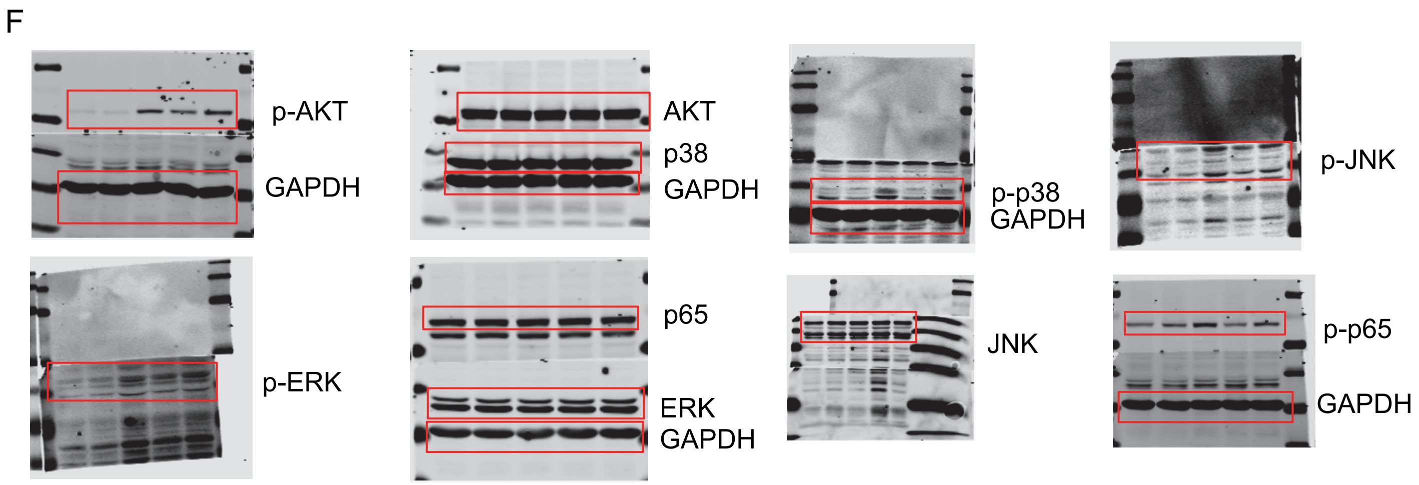

Full unedited gel for Figure 8E

Jiang et al., Supplemental Fig. 8

G

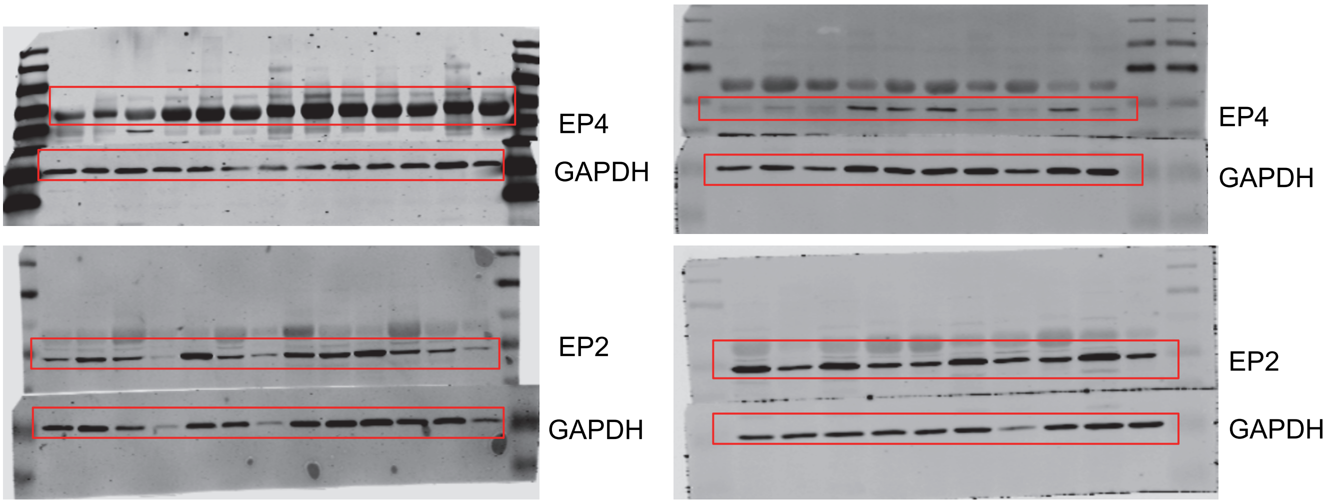

Full unedited gel for Supplemental Fig. 1D

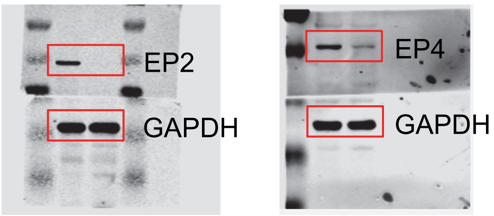

Full unedited gel for Supplemental Fig. 1E

H

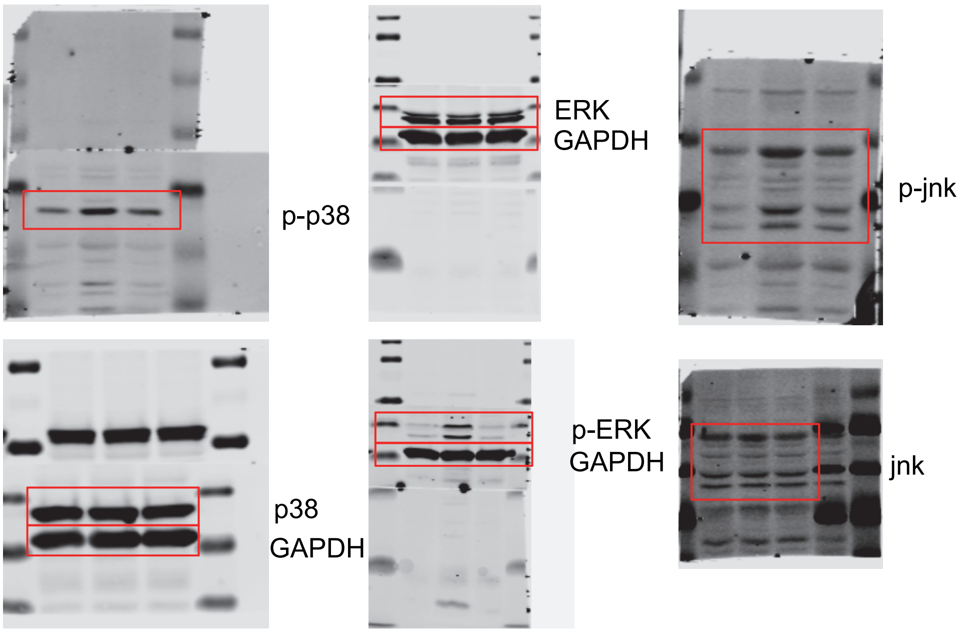

Full unedited gel for Supplemental Fig. 7B

I

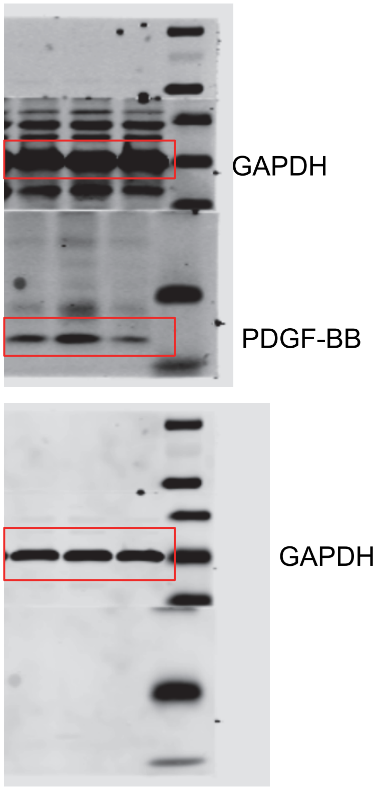

Full unedited gel for Supplemental Fig. 7D
